# Complement Proteins as Soluble Pattern Recognition Receptors for Pathogenic Viruses

**DOI:** 10.3390/v13050824

**Published:** 2021-05-02

**Authors:** Valarmathy Murugaiah, Praveen M. Varghese, Nazar Beirag, Syreeta DeCordova, Robert B. Sim, Uday Kishore

**Affiliations:** 1 Biosciences, College of Health, Medicine and Life Sciences, Brunel University London, Uxbridge UB8 3PH, UK; Valarmathy.Murugaiah2@brunel.ac.uk (V.M.); Praveen.MathewsVarghese@brunel.ac.uk (P.M.V.); Nazar.beirag@brunel.ac.uk (N.B.); syreeta.decordova@yahoo.com (S.D.); 2 Department of Biochemistry, University of Oxford, South Parks Road, Oxford OX1 3QU, UK; edith.sim@pharm.ox.ac.uk

**Keywords:** innate immunity, complement system, complement evasion, DNA viruses, RNA viruses, retroviruses, cytokine storm

## Abstract

The complement system represents a crucial part of innate immunity. It contains a diverse range of soluble activators, membrane-bound receptors, and regulators. Its principal function is to eliminate pathogens via activation of three distinct pathways: classical, alternative, and lectin. In the case of viruses, the complement activation results in effector functions such as virion opsonisation by complement components, phagocytosis induction, virolysis by the membrane attack complex, and promotion of immune responses through anaphylatoxins and chemotactic factors. Recent studies have shown that the addition of individual complement components can neutralise viruses without requiring the activation of the complement cascade. While the complement-mediated effector functions can neutralise a diverse range of viruses, numerous viruses have evolved mechanisms to subvert complement recognition/activation by encoding several proteins that inhibit the complement system, contributing to viral survival and pathogenesis. This review focuses on these complement-dependent and -independent interactions of complement components (especially C1q, C4b-binding protein, properdin, factor H, Mannose-binding lectin, and Ficolins) with several viruses and their consequences.

## 1. Introduction

**Figure 1:**
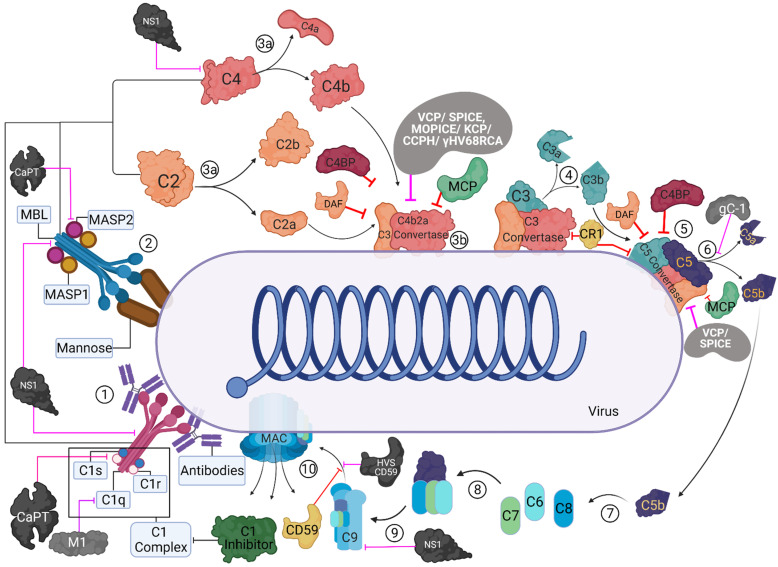
Activation and regulation of the classical and lectin pathways and their targeting by virally encoded molecules. In the classical pathway (CP), C1 complex recognizes the antigen-antibody complexes present on the viral surface (1). In the lectin pathway (LP), MBL/ficolin-MASP complexes can recognise other carbohydrate patterns on the surfaces of viruses (2). Upon activation, these complexes can cleave C4 and C2 (3a) that can lead to the formation of C4bC2a (CP/LP C3 convertase) (3b). The C3 convertase further cleaves C3 into C3b and C3a; C3b is known to opsonise the viral surfaces, whereas C3a can lead to an enhanced acquired immune responses (4). C3b-C3 convertase interaction can generate C5 convertase (5), which cleaves C5 into C5b and C5a (6). C5b further interacts with C6 and C7 (C5b-7) (7) that can bind to the viral surface, while C5a induces further infiltration. C5b-7 then binds to C8, which can generate C5b-8 that penetrates the membrane (8). Finally, the C9 binds to the C5b-8 and results in MAC formation leading to the virolysis (10). These activation pathways are regulated at different steps by host complement regulators such as C1 inhibitor, C4b-binding protein (C4BP), complement receptor 1 (CR1; CD35), membrane cofactor protein (MCP; CD46), decay-accelerating factor (DAF; CD55), and CD59. Viral proteins that target these pathways are: Vaccinia virus complement control protein (VCP), Smallpox inhibitor of complement enzymes (SPICE), Monkeypox inhibitor of complement enzymes (MOPICE), Kaposi’s sarcoma-associated herpesvirus inhibitor of complement activation (KCP), Murine gamma-herpesvirus 68 regulator of complement activation (γ-HV68 RCA), Herpesvirus saimiri complement control protein homologue (CCPH), Herpesvirus saimiri CD59 homologue (HVS CD59), Flavivirus non-structural protein 1 (NS1), HSV-1 glycoprotein C (gC-1), human astrovirus coat protein (CoPt), and Influenza virus matrix protein 1 (M1). These are identified as black/grey protein with white text, and pink inhibitory arrows mark the regulator they inhibit.

The innate immune system is characterised by its ability to distinguish between “self” and “non-Self”. The complement system plays a crucial part in the innate immune surveillance against viruses through several mechanisms that prevent host viral infection. It can be activated through three pathways: the classical, the alternative, and the lectin, depending upon the recognition subcomponents and the ligand that trigger its activation. The classical pathway is activated (Figure 1) by either direct binding of complement component C1q to the invading pathogen’s surface, or the binding of IgM, IgG1, and IgG3 to the antigen’s surface and the subsequent binding of C1q to this immune complex. The binding of C1q to either antibodies or pathogen surface triggers the autoactivation of serine protease, C1r, which subsequently cleaves and activates another serine protease, C1s [1]. This generates a C1-complex consisting of one molecule of C1q and two molecules each of C1r and C1s. The C1 complex then cleaves C4 and C2, generating C4a, C4b, C2a, and C2b. The C4b and C2a bind to form the C4b2a complex, the C3-convertase [2,3,4].

The lectin pathway is a homologue of the classical pathway. It is triggered by the binding of mannan-binding lectin (MBL) and ficolins to carbohydrate patterns on the pathogen surface or carbohydrate structures on antibodies, including the common IgG glycosylation variant IgG-G0 and polymeric IgA [1,5,6,7,8]. In serum, MBL is found complexed with homologues of C1r and C1s, called MBL-associated serine proteases (MASPs) [1,9]. Upon MBL binding to a target, MASP-1 and MASP-2 autoactivate independently. MASP-2 cleaves C4 and C2, triggering the formation of the C3-convertase similar to the classical pathway.

**Figure 2:**
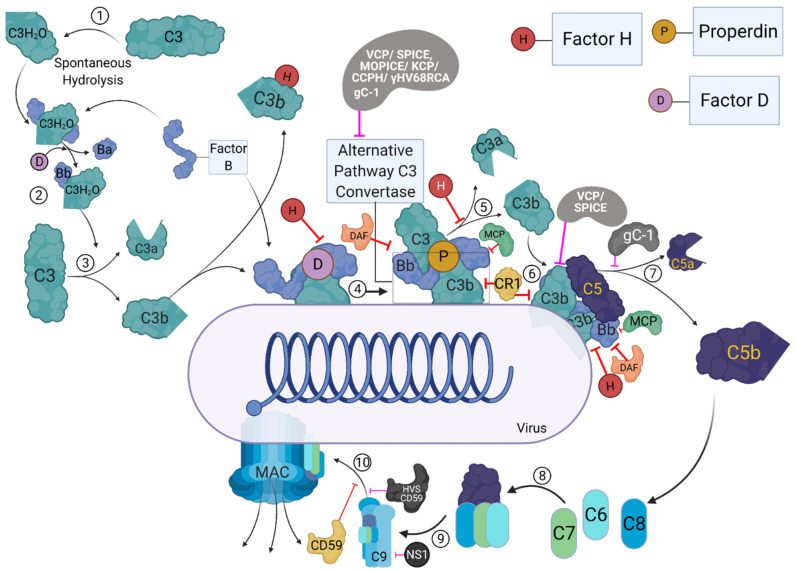
The activation and regulation of the alternative pathway and its targeting by virally encoded molecules. During the process of alternative pathway (AP), native C3 by H_2_O is spontaneously hydrolysed, resulting in the formation of C3b like C3 [C3(H_2_O)] (1). C3(H_2_O) can bind to factor B (FB), and upon cleavage by factor D (FD), which forms the initial AP-derived C3 convertase (2). The C3 convertase can cleave C3 into C3b and C3a (3). The C3b then binds to the viral surfaces, and trigger the formation of surface bound C3bBb, with the involvement of FB and FD (4). The surface bound C3bBb can then initiate the amplification loop of the AP (5), causing deposition of C3b molecules on to viral surfaces. C3b can combine with pre-existing AP-derived C3 convertase, which leads to the formation of C5 convertase (6). C5 convertase cleaves C5 into C5b and C5a (7). C5b further interacts with C6 and C7 to form C5b-7 (8), which can bind to the surfaces of viruses, while C5a acts as an anaphylatoxins. C5b-7 then binds to C8 which can generate C5b-8 that penetrates the membrane (9). Finally, the C9 binds to C5b-8, resulting in MAC formation (10). The activation steps are regulated at different steps by host complement regulators such as complement receptor 1 (CR1; CD35), membrane cofactor protein (MCP, CD46), decay-accelerating factor (DAF; CD55), factor H (FH), and CD59. Viral proteins that target these pathways are: Vaccinia virus complement control protein (VCP), Smallpox inhibitor of complement enzymes (SPICE), Monkeypox inhibitor of complement enzymes (MOPICE), Kaposi’s sarcoma-associated herpesvirus inhibitor of complement activation (KCP), Murine gamma-herpesvirus 68 regulator of complement activation (γ-HV68 RCA), Herpesvirus saimiri complement control protein homologue (CCPH), Herpesvirus saimiri CD59 homologue (HVS CD59), Flavivirus non-structural protein 1 (NS1), and HSV-1 glycoprotein C (gC-1). These viral proteins are identified as black/grey proteins with white text, and pink inhibitory arrows mark the regulator they inhibit.

A distinct mechanism (Figure 2) activates the alternative pathway. It has both antibody-dependent (IgG) and antibody-independent modes of activation. It is continuously activated by the spontaneous hydrolysis of C3 into C3(H_2_O) (also known as C3i). C3i binds with factor B, a serine protease, forming a complex, called C3iB. C3iB enables factor D to cleave factor B to Bb, forming C3iBb. This newly formed C3iBb cleaves C3 to form C3b and C3a. The C3b formed binds to the pathogen surface where they further bind more factor B, which are then cleaved by factor D to form C3bBb, similar to the C3 convertase of the other two pathways.

The three complement pathways converge on C3 convertase, which is considered the complement system’s central component [10]. The C3 convertase promotes the cleavage of C3 into C3a and C3b. C3b then binds with C4b2a (of the classical and lectin pathways) complex or C3bBb (of the alternative pathway) converting them into classical/lectin or alternative pathway C5 convertase, respectively. C5 is the initiator of the complement system’s effector terminal phase, which is similar for all three pathways. The C5 convertases cleave C5 at the position Arg751-Leu752 on the α chain to form C5a and C5b [11]. The C5b produced acts as a nucleus for the assembly of the membrane attack complex (MAC) [12]. The C5b interacts with C6, and the C5b6 complex is further stabilised by binding to C7. The binding of C7 also exposes transient lipid-binding sites, which allow the complex to bind to the cell membrane. This binding does not harm the cell but marks it for further assault. The C5b-7 complex then interacts with C8, which forms the tetrameric complex C5b-8, promoting binding and polymerization of 10 to 16 molecules of C9. The complement system’s terminal phase concludes with the insertion of this C5b-9 complex to the microbial surface. This leads to the opsonisation and subsequent lysis of the microbe.

The C3a and C5a generated during complement activation are anaphylatoxins. C5a is known to bind to cells expressing C5aR and C5L2, while C3a is known to bind to cells expressing C3aR [11,13,14]. By interacting with C5aR and C3aR, these anaphylatoxins induce smooth muscle contraction and increase vascular permeability [15,16]. C3a and C5a have been reported to trigger oxidative burst in macrophages, neutrophils, and eosinophils [17,18,19]. These anaphylatoxins can also induce the release of histamine from basophils and mast cells [20,21]. In B cells and monocytes, C3a modulates the production of IL-6 and TNF-α. C3a can also act as a chemoattractant for mast cells [22,23,24]. C5a is known to act as a chemoattractant for macrophages, neutrophils, activated B and T cells, basophils, and mast cells [15]. These actions make the anaphylatoxins potent mediators of inflammation [25].

Dysregulated activation of the classical and lectin pathways during ischemia-reperfusion injury is known to cause necrosis, apoptosis, and possibly autophagy of the tissue, thus potentially causing permanent tissue or organ damage [26]. Common effector mechanisms that depend on C5a and C5b-9 are responsible for the tissue damage [27]. Similarly, the dysregulation of the non-discriminatory C3b deposition and amplification by the alternative pathway and anaphylatoxin production can damage host cells rapidly [28]. Hence, the complement system is kept in check by various regulatory proteins to reduce such undesired inflammatory responses and tissue damage. In the alternative pathway, the spontaneously generated C3b in the absence of an antigen is sequestered by factor H and factor I [29] (Figure 2). The covalent binding of C3b to the microbial surface protects the C3b from factor H-mediated inactivation [29]. Factor H also promotes the decay of C3bBb convertases by dissociating Bb from the proconvertase [30,31]. It also acts as a co-factor for factor I (fI)-mediated cleavage of C3b, preventing the formation of new convertase [6,32]. C4b-binding protein (C4BP) and C1 inhibitor (C1-INH) regulate the classical and lectin pathways. C4BP regulates complement activation by controlling C4b-mediated reactions. These include promoting the dissociation of C4bC2a convertases, inhibiting the formation of C3 and C5 convertases, and acting as a co-factor for fI-mediated cleavage of C4b [33,34,35]. C1-INH is a serine protease inhibitor that inhibits C1r and C1s of the classical pathway and MASPs of the lectin pathway [9] (Figure 1). Other regulators of the complement system (Figure 1 and Figure 2) include intrinsic membrane proteins found on host cells, such as decay-accelerating factor (DAF/CD55), membrane co-factor protein (MCP/CD46), complement receptor 1 (CR1/CD35), and CD59 (protectin) [29]. The DAF regulates the classical and alternative pathways by destabilizing their C3/C5 convertases (termed decay-accelerating activity) [36], while the MCP functions as a cofactor for fI–mediated cleavage of C3b and C4b (termed cofactor activity) [37]. CR1 is known to have both decay-accelerating and cofactor activities, while CD59 blocks the C9 association with C5b-8, preventing the formation of the MAC on host cells [38,39].

This review focuses on the complement activation-dependent and independent functions of complement components (especially C1q, C4BP, properdin, factor H, MBL and Ficolins) as soluble pattern recognition receptors for several viruses (Table 1).

## 2. Role of the Complement System during Viral Infection: Viral Evasion Mechanisms

The complement system employs multiple mechanisms that inhibit the viral infection of the host. Complement activation neutralises viruses via (a) viral opsonisation by complement components; (b) virolysis, which occurs when the MAC produces holes on the viral membrane; and (c) the production of anaphylatoxin [40,41,42]. The viral neutralisation occurs due to the deposition of complement proteins on viral surfaces, which can block virus–host receptor interactions. It can further cause aggregation of viral particles, and trigger an anti-viral state, as well as by enhancing phagocytosis [43,44,45]. Enveloped viruses such as alphaviruses, coronaviruses, herpesviruses, and retroviruses are susceptible to lysis by the MAC [46]. In addition, the anaphylatoxins produced during the complement activation lead to pro-inflammatory responses and enhanced phagocytosis [25]. Complement activation is also known to induce a Th1 response, modulate Treg and Th17 responses, prolong B-cell memory, and significantly increase antigen-specific antibody titres [47,48,49]. Thus, complement activation also enhances an adaptive immune response against the virus.

The complement system’s importance against pathogens is best exhibited by the coevolution of the pathogens with the hosts. Viruses mainly achieve complement evasion (Figure 1 and Figure 2) by either binding to complement proteins (Table 1) or expressing homologues of host complement control proteins (Table 2).

Gamma herpesviruses such as Murine gamma-herpesvirus 68 (γ-HV68), Herpesvirus saimiri (HVS), and Kaposi’s sarcoma-associated herpesvirus (KSHV) are known to encode homologues of regulator of complement activation (RCA). The complement control protein homologue (CCPH) produced by the HVS shares a similar global structural layout with DAF and MCP. CCPH is known to inhibit complement activation by accelerating decay of the C3 convertase and by its FI cofactor activity for the cleavage of C4b and C3b, thus inhibiting all three complement pathways [50,51]. In addition to the RCA homologue, HVS is also known to encode a homologue of CD59, the inhibitor for the MAC complex [52]. The KSHV encoded KSHV complement control protein (KCP), another RCA homologue of DAF and MCP, inhibits complement activation by accelerating the decay of the classical pathway C3-convertase, but not the C3 convertase of the alternative pathway, and inactivating C3b and C4b through FI-mediated cleavage activity [53,54,55,56]. In vitro analysis of the γ-HV68 RCA, which is also a homologue of DAF and MCP, has revealed that it blocks the deposition of C3 on zymosan beads, suggesting it acts on the C3 convertase to block the complement system [57,58].Poxviruses also encode complement regulatory proteins such as variola virus inhibitor of complement enzymes (SPICE), the vaccinia virus complement control protein (VCP), the monkey pox virus inhibitor of complement enzymes (MOPICE), and the ectromelia virus inhibitor of complement enzymes (EMICE) [9]. VCP produced by the vaccina virus and the SPICE protein, an orthologue of VCP, produced by smallpox causing variola virus, share homology with MCP and are shown to have decay-accelerating activity against and cofactor activity, helping the viruses evade the complement system [59,60,61,62,63]. In addition to expressing VCP, extracellular enveloped vaccinia virus is also known to incorporate host complement regulators CD46, CD55, and CD59 into their outer envelope [64]. Another VCP orthologue, MOPICE that is produced by the monkey pox virus, has been shown to possess the cofactor activity but it lacks the decay-accelerating activity.

The coat protein of astrovirus (CoPt) inhibits the activation of the classical and lectin pathways by binding C1q and MBL [65,66]. CoPt is shown to share limited sequence homology with human neutrophil defensin-1, a known inactivator of the classical and lectin pathways [67]. CoPt helps the virus evade the classical pathway by disassociating C1s from C1q and preventing the cleavage of C1s to its active form, thereby inhibiting C1 activation [65,66]. CoPt also inhibits the lectin pathway in a similar manner by binding to MBL using residues critical for MASP-2 binding [66]. Furthermore, CoPt can also inhibit C5a production [66].

Another complement evasion mechanism used by viruses (Figure 1 and Figure 2) involves production of non-homologous viral proteins that can interact with or recruit host complement regulators (Table 2). Alpha-herpesviruses such as Herpes Simplex Virus (HSV) Type 1 and HSV Type 2 are known to encode envelope surface glycoprotein C (gC) that protects them against complement activation [58]. gC-1, produced by HSV-1, has two complement-interacting domains, a C3-binding region in the centre of the molecule and an amino-terminal domain that interferes with C5 and properdin binding to C3b [68,69,70]. In vivo studies in murine models have demonstrated that the C3 binding domain plays a more critical role in HSV virulence [68]. While both the domains contribute to virulence, the deletion of the C3 domain attenuates the virus infection ability to levels similar to viral mutants lacking both the domains [68]. gC-2, produced by HSV type 2, and gC-1 have been reported to bind to native C3, C3b, iC3b, and C3c [70,71,72,73]. gC-1 is known to inhibit complement activation by blocking the interaction of C3b with properdin and C5, accelerating the decay of the alternative pathway C3 convertase [69]. However, gC-2 lacks the domain that blocks properdin and C5 interaction with C3b [70]. This suggests that the mechanism of complement subversion by gC-2 is different compared to that of gC-1 [70].

Flaviviruses, which cause diseases such as Dengue (DENV), Zika, and Yellow fever (YFV), produce a non-structural protein 1 (NS1), which is a secreted non-structural glycoprotein that accumulates in the blood and is displayed on the surface of infected cells. The West Nile Virus (WNV) NS1 has been shown to bind factor H, which retains its co-factor activity [74]. Simultaneously, cell surface-associated NS1 reduces the deposition of C3b and the C5b–C9 membrane attack complex [74,75,76]. NS1 of DENV, WNV, and YFV also helps the viruses evade neutralisation by the complement system by interacting with C4 and C1s [77]. The NS1-C4 interaction reduces the classical C3 convertase formation as well as the deposition of C4b and C3b on cell surfaces [77]. Similarly, NS1 regulates both the classical and the lectin pathways by recruiting C4BP [78]. This is accomplished by C4BP acting as a cofactor for FI, mediating the inactivation of C4b in solution as well as on the plasma membrane of infected cells [78]. DENV NS1 has also been shown to bind to clusterin [79] (and vitronectin), a regulatory protein that hinders the insertion of the MAC into membranes and binds the terminal complement proteins [80]. This interaction protects the virus from virolysis by inhibiting C9 polymerization [80].

## 3. C1q Exploiting Viral Evasion Mechanisms

Human C1q can recognise and bind a variety of self and non-self ligands and regulate a range of homeostatic functions such as clearance of immune complexes, pathogens (including viruses, bacteria, and fungi), and necrotic and apoptotic cells [81]. C1q is a primordial innate immune molecule and a first subcomponent of the C1 complex that recognises the IgG-or IgM containing immune complexes [82]. C1q is a 460 kDa protein, comprising of 18 polypeptides chains (including 6A, 6B, and 6C), where each C1q chain is composed of a short N-terminal region, a triple-helical collagen region, and a C-terminal globular (gC1q) domain [81,83]. Although the liver secretes most of the C1q, production of C1q by macrophages, adherent monocytes, and immature DCs has also been reported [84,85,86]. In addition, C1q is also abundant in the microenvironment of various tumour tissues, where it is considered to be tumorigenic on its own, without recruiting classical complement cascade [87].

The viral neutralizing activity of C1q against Influenza A Virus (IAV) has been studied using in vitro models. C1q was found to enhance haemagglutinin (HA)-specific monoclonal antibody-mediated inhibition of IAV attachment to host cells (>100-fold) at the cell-binding stage [88]. Furthermore, matrix protein 1 (M1), a conserved multifunctional protein of IAV, was found to interact with the globular region of C1q A chain [89]. This interaction between M1 and C1qA takes place through the N-terminal domain of the M1 protein [89]. The M1 protein was able to block C1q A chain interaction with heat-aggregated IgG, thereby inhibiting haemolysis as well as preventing the complement-mediated neutralization of IAV in vitro [89].

C1q interaction with retroviruses involves the globular region of C1q and envelope glycoproteins of several viruses, including gp41 and gp120 of HIV-1, p15E of murine leukaemia virus (MuLV), and gp21 of human T lymphotropic virus (HTLV)-1 [90]. C1q via its gC1q domain, as well as its globular head receptor, gC1qR, can interact with gp41 of HIV-1 [91,92,93], primarily via the C1q A chain [94], in a similar way to C1q interaction with IgG [90,95]. Similar to IgG, aggregates formed by gp41 [92] can further lead to an increased C1 complex activation. Thus, gp41 can trigger the classical pathway on the surface of virus-infected cells in an antibody-independent manner [96]. Furthermore, gp120 of HIV-1 can also enhance antibody-mediated complement activation through binding C1q or mannan-binding lectin (MBL) of the lectin pathway [97,98,99]. Additionally, C1q- or C3-deficient human serum from uninfected individuals as a source of complement does not trigger any anti-viral activity against HIV-1, suggesting that the classical pathway contributes mainly to the complement activation against HIV-1 [100].

C1q can suppress DC-SIGN-mediated transfer of HIV-1 to activated peripheral blood mononuclear cells; however, the recombinant form of globular head A, B, and C modules of C1q do not [101]. The protective activity of C1q was negated by the addition of gC1qR by enhancing DC-SIGN mediated HIV-1 transfer. It is possible that C1q presence can play a protective role by blocking access of gp120 to DC-SIGN. Furthermore, gC1qR, as an inhibitor of HIV-1 infection on its own, can block the CD4-gp120 interaction, thus, preventing viral entry ([102]. However, other studies have suggested that soluble gC1qR alone can also suppress HIV-1 production in MT-4/H9 human T cell lines and macrophages infected with HIV-1_Ba-L_ or HIV-1_IIIB_ [102]. Thus, suppression of HIV-1 production was enhanced following pre-incubation of gC1qR with the target cell lines before viral challenge, indicating that the ability of gC1qR to interfere with viral entry occurs through the interaction between CD4 and gp120 of HIV-1 [102]. Furthermore, binding interactions of gC1qR with several viral ligands, such as HCV core protein [103], rubella viral capsid protein [104], adenovirus core protein V [105], and EBNA-1 of Epstein-Barr virus [106], have also been reported. The gp41 of HIV-1 can also engage with gC1qR on CD4^+^ T cells to trigger an NK ligand expression through PI3K/PIP3 pathway, suggesting that gC1qR can act as a receptor for HIV-1 [91]. Fausther-Bovendo et al. have reported that gC1qR can serve as a receptor for a specific motif of HIV-1 gp41, known as 3S. 3S is highly conserved in HIV-1 isolates, which plays a crucial role in inducing cell surface-expression of NKp44L on CD4^+^ T cells [107]. NKp44L is a known cellular ligand for the natural cytotoxicity receptor NKp44 [108], and it can render CD4^+^ T cells susceptible to autologous NK lysis. In addition, NKp44L expression is significantly correlated with an enhanced viral load as well as declined CD4 cell count [107]. Furthermore, the 3S motif can trigger PI3K/PIP3 pathway, which is crucial for the 3S-mediated signalling that results in the translocation of NKp44L to the cell surface [91]. Braun et al. have previously reported that the binding of internalin B of *Listeria monocytogenes* to gC1qR activates PI3K signalling [109].

Co-infecting MT4 or SLB1 cells with HIV-1 and HTLV-I can recruit C1q and form active C1 on the cell surface [110], leading to complement activation [110]. C1q binding to HTLV-1 has also been confirmed by another study using the cell-free virus HTLV-1 lysates. C1q was able to inhibit the infectivity of cell-free HTLV-1 [111]. The same study has also reported that C1q can bind an extramembrane region of the HTLV-I gp21 (residues 400–429) [111], a region that is crucial for syncytium formation [112]. C1q can also bind MuLV p15E directly and activate the classical pathway, resulting in virolysis without the involvement of antibodies [113]. Furthermore, purified C1q can directly bind to Chandipura virus (CHPV) but the binding interaction does not affect the viral infectivity; CHPV neutralisation requires C1q-reconstituted serum [114].

## 4. Viral Evasion Strategies Exploiting C4b Binding Protein

C4BP is a 570 kDa spider-like glycoprotein, made up of 7 identical 70 kDa α-chains and a 45 kDa β-chain, linked together by a central core [115]. The α- chains and β-chain contain eight and three Complement Control Protein (CCP) domains, respectively [33]. These CCP modules are composed of ~60 amino acids and form a compact hydrophobic core surrounded by five or more β-strands organized into β-sheets [116]. C4BP functions as a regulator of the classical and lectin pathways by controlling C4b-mediated reactions [117], inhibiting the formation of C3 and C5 convertases, accelerating the decay of the convertases, and by acting as a co-factor for FI, which cleaves and thereby inactivates fluid phase and cell-bound C4b [34,35,116,118].

Flaviviruses are known to limit complement activation by binding C4BP through their NS1 protein; the bound C4BP inactivates soluble or membrane-bound C4b [78]. The binding of flavivirus NS1 to C4BP is mediated through multiple CCP domains (CCP2-5 and CCP8) of the C4BP α-chain [78]. Furthermore, the involvement of CCP domain 8, which is near the C-terminal oligomerisation domain of C4BP, could affect the conformational structure of C4BP. Hence, the absence of CCP8 (via recombinant deletion) could affect the accessibility of CCP2-5 for flavivirus NS1 [78].

The Hepatitis B virus is known to cause hepatocarcinogenesis via its X protein (HBx) [119]. It has been reported that HBx protects hepatoma cells from complement attack by increasing the surface expression of complement regulatory proteins such as CD46 and CD59 [120,121]. It is also known to up-regulate C4BPα through transcription factor Sp1 in hepatoma cells, thereby inhibiting complement activation [119].

C4BP can also interact directly with pathogens without needing to deal with C4b deposition. For example, C4BP is known to facilitate the uptake of adenoviruses by hepatocytes via its interaction with cell surface heparin–sulphate proteoglycans (16). C4BP was also found to reduce hepatic toxicity after systemic application of adenoviruses vector [122]. A chimeric disulphide-bound homo-octameric protein, sCD46-C4BPα (generated by the fusion of the C4BPα bundle domain ectodomain of CD46), has been shown to control measles virus infection in vitro as well as in CD46 expressing transgenic mice [123]. A 2-fold increase in anti-viral activity was observed by the fusion protein when compared to monomeric sCD46. The mechanism probably involves: (i) the competition between cell surface CD46 receptor, which is needed for the binding and fusion of measles virus and sCD46-C4BPα; and (ii) the irreversible conformational change of the fusion protein induced by the simultaneous binding to multiple measles virus envelope glycoprotein, hemagglutinin [123].

**Figure 3:**
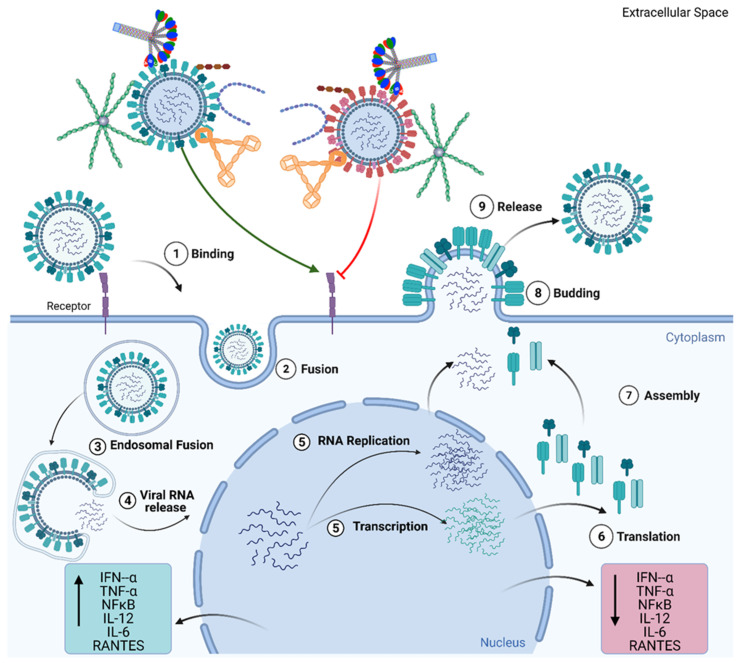
Complement Independent functions of Complement Regulators. Viral infection begins with the attachment of the virus to the epithelial cell surface via cell surface receptors (1) and the internalisation of the virion through endocytosis and fusion (2). Post endocytosis, viral RNA is released into the cytoplasm (3,4), from where it is transported into the nucleus. In the nucleus, the viral RNA undergoes replication and transcription (5). The transcribed mRNA is translated to viral proteins (6). This is followed by the assembly of the virion and subsequent release of the virion from the cell (7,8,9). C1q, C4BP, Properdin, factor H, and VCP have individually been shown to inhibit the entry of viruses, such as the H1N1 subtype of the Influenza A Virus (IAV), (represented by red virion) into the cell and downregulate inflammatory cytokines and chemokines (TNF-α, IL-6, IL-12, NF-κB, RANTES). However, these complement regulators individually have also been implicated in promoting viral entry, as seen in the case of H3N2 subtype of IAV, and promoting the inflammatory response by upregulating cytokine and chemokines (TNF-α, IL-6, IL-12, NF-κB, and RANTES). These mechanisms of modulating viral entry in a subtype specific manner, occur in the absence of other complement factors and immune cells, suggesting complement independent viral infection modulating activity for these complement regulatory proteins.

Recently, C4BP has been shown to differentially modulate the efficacy of IAV entry and replication in human adenocarcinoma alveolar basal epithelial cells, A549, in a strain-dependent and complement-independent manner (Figure 3) [124]. C4BP can bind IAV envelope proteins: Haemagglutinin, Neuraminidase, and Matrix protein 1 via multiple sites in CCP domains 1–2, 4–5, and 7–8 of its α-chain. In the case of the H1N1 subtype of IAV, C4BP was found to restrict viral entry and infection in A549 cells. However, C4BP promoted viral entry and infection in the case of the H3N2 subtype. Furthermore, C4BP downregulated mRNA levels of pro-inflammatory IFN-α and IL-12 (and NF-κB) in the case of H1N1. However, it promoted a pro-inflammatory immune response by upregulating IFN-α, TNF-α, RANTES, and IL-6 in the case of H3N2 [124].

## 5. Involvement of Properdin in Anti-Viral Immune Response and Viral Evasion

The human properdin gene *CFP* (Complement factor P) encodes for properdin protein [125], which circulates in serum as cyclic polymers formed by head-to-tail association in cyclic dimers, trimers, and tetramer structures in a 26:54:20 ratio, respectively, with plasma concentration of 22–25 µg/mL [126,127,128]. An aglycosylated monomer of properdin has a mass of 53 KDa on an SDS-PAGE gel under reducing conditions [126]. Properdin is composed of seven repetitive, non-identical motifs of 60 amino acids, each of which are called thrombospondin repeats (TSR), which are TSR0 to TSR6; the N terminal module, TSR0, is truncated while TSR4 and 5 are crucial for binding to C3bBb, and hence, stabilizing C3 convertase [129,130]. Properdin is the only positive regulator of the alternative pathway [131]. It prevents the dissociation of Bb from C3b by increasing the half-life of this complex (C3bBb) from 90 s up to 10-fold [30]. Properdin is synthesized and/or secreted by a wide range of immune cells, including mast cells, macrophages, monocytes, T cells, dendritic cells, and neutrophils [132,133,134,135,136]. Deficiency of human properdin increases the risk of *Neisseria meningitidis* infection [137].

Properdin can exert immune functions in a complement-independent manner, including its ability to bind to microbial targets *Neisseria meningitidis* lipopolysaccharide and *Chlamydia pneumonia* [138]. Properdin ligands include DNA, sulfatides and glycosaminoglycans; these interactions are crucial in phagocytosis-driven removal of unwanted debris and in avoiding harmful inflammation via elimination of apoptotic/necrotic cells [139] via direct binding as an opsonin [140,141]. In addition, properdin can act as a PRR molecule against infectious agents such as *Neisseria* by binding to lipo-oligosaccharide (LOS) [138,142].

Kaposi’s sarcoma-associated herpesvirus (KSHV) infection induces cell surface expression of properdin in the infected endothelial cells, which is essential for complement activation during *de novo* KSHV infection [143]. Dengue virus-infected endothelial cells were found to have a high-level induction of properdin and factor B, providing a direct means for dengue virus-infected endothelial cells to enhance complement activation and C3a and C5a production [144]. Properdin is also known to bind gp41 and gp 120, subunits of the HIV-1 envelope [145]. Sites in gp120 seem to be involved around amino acid 100–129 in properdin interaction. Thus, properdin may contribute to inhibiting HIV-1 from binding to CD4 receptor as well as the fusion of viral envelope with the cell membrane [145]. Properdin has been shown to act as a ligand for the NKp46 receptor on NK cells, resulting in up-regulation of the XCL1 chemokine gene, the chemokine (also known as lymphotactin), leading to antiviral activity such as blocking HIV-1 attachment and entry into host cells [146,147,148,149]. Properdin is expressed by CD8^+^-T cells, which play a significant role in the elimination and clearance of viral infection [134,150]. Additionally, it has been shown that neutrophils are among the first responder to IAV infection in the lung, which can cause the release of properdin due to pro-inflammatory cytokines such as IFN type 1 [133,135,151]. Furthermore, neutralisation assays conducted on pseudotyped lentiviral particles expressing IAV envelope proteins (matched H1+N1 or unmatched H3+N2) and replication kinetic analysis on H1N1 or H3N2 infected A549 cells revealed that properdin treatment modulated IAV entry and subsequently IAV replication in a subtype-dependent manner (Figure 3) (Varghese et al., Unpublished Data). However, better insights into the role of properdin for various common human viral infections are still poorly studied.

## 6. Factor H

The alternative pathway plays an important role in the protection against viruses. Activation of the alternative pathway is limited on the self-cells by various negative regulators, including FH, the primary soluble regulator. In the presence of sialic acid found on self-cells, the affinity of FH for surface-bound C3b is increased and allows FH to differentiate between self and non-self-cells [58,152,153]. FH can block activation of the alternative pathway by accelerating the decay of the alternative pathway C3 convertase (C3bBb), acting as a co-factor for factor I mediated cleavage of C3b, and competing with factor B for C3b binding [154,155,156]. FH is composed of 20 complement control protein (CCP) domains with CCP 1-4 encompassing functional activity and the ability to bind C3b has been mapped to CCP 19-20, 7-15, and 1-4 regions [31].

FH is part of the FH family, a group of highly related multifunctional protein primarily synthesised in the liver, and include FH, the spliced variant FH-like protein 1 (FHL-1) and FH-related proteins (FHR) 1–5 [157,158]. These proteins share conserved common structural elements and display overlapping roles in complement regulation, particularly FHL-1 and FHR-5 [157,158,159]. Given the FH family proteins’ ability to act as negative regulators (with a few exceptions) of the alternative pathway, it is likely that they play an important role during viral infections. Over time, some viruses have developed the ability to manipulate the function of FH as an inhibitor of the alternative pathway to enable them to escape complement destruction [31]. Sindbis virus is an enveloped Alphavirus from the Togaviruses family. The Sindbis fever is characterised by arthralgia, rash, and malaise. It can acquire sialic acid during the process of budding off the host cells, which inversely correlates with the activation of the alternative pathway. FH recognises the virus as a host cell due to the increased presence of sialic acid and blocks complement-mediated virolysis [31]. The ability of FH to recognise sialic acid from the virus is crucial in Sindbis virus resistance to the alternative pathway [152,160]. HIV-1 exploits the complement regulatory role of FH for its benefit. FH is recruited on to the surface of HIV-1, providing a mechanism for the virus to escape complement activation [161]. HIV-1 virions treated with FH-deficient serum and anti-HIV-1 antibodies were lysed in a complement-dependent manner. FH binding to gp120 and gp41 of HIV-1 at high local concentrations protects the virus from complement destruction [161,162].

West Nile virus (WNV) is an enveloped RNA Flavivirus; the RNA is translated in the host cytoplasm as a polyprotein and then cleaved into structural and non-structural (NS) proteins by the virus- and host-encoded proteases [163,164]. WNV NS-1, secreted at high levels in infected patients, binds to FH evasion [58,74], and promotes FH to act as a co-factor for FI-mediated cleavage of C3b, preventing activation of the alternative pathway [74]. Recently, Murugaiah et al. have reported complement-independent modulation of IAV infection by FH in a subtype-dependent manner (Figure 3) [165]. FH accomplished the IAV subtype dependent entry modulation via interaction with glycoproteins HA, NA, and M1. Modulation of viral entry by r FH was evident by downregulation (−4 log_10_) of M1 of IAV in H1N1 subtype infected A549 cells, while upregulation (2 log_10_) was seen in H3N2-infected cells. FH was found to trigger anti-inflammatory responses in H1N1 infected A549 cells while provoking pro-inflammatory responses in the case of the H3N2 subtype. mRNA expression levels of TNF-α, IL-12, IL-6, and IFN-α were upregulated, while RANTES was downregulated in H1N1-infected A549 cells treated with FH at 6 h post-infection. In the case of the H3N2 subtype, enhanced mRNA levels of these pro-inflammatory cytokines were observed. The same study also revealed that FH could act as an entry inhibitor for the H1N1 subtype, as evident by a reduction in luciferase reporter activity in MDCK cells transduced with H1N1 pseudotyped lentiviral particles [165].

## 7. Human Mannan-Binding Lectin (MBL)

Human mannan-binding lectin (MBL) is a soluble, Ca^2+^-dependent pattern recognition innate immune molecule, which acts as a potent opsonin against invading pathogens [166]. MBL is the recognition subcomponent of the lectin pathway through association with MBL-associated serine proteases (MASPs) [167,168]. The overall structure of MBL comprises of oligomers of trimeric subunits, composed of an N-terminal cysteine-rich domain, triple-helical collagenous region (made up of Gly-X-Y repeats), α-helical coiled-coil trimerizing neck region, and a carbohydrate recognition domain (CRD) [169,170,171]. Human MBL is primarily produced by hepatocytes and secreted into the bloodstream [172,173,174]. However, a reduced MBL expression has been reported in mammalian muscle tissues and the brain [175]. Humans and chimpanzees present with only one form of MBL [176], but two forms of MBL (MBL-A and MBL-C) were reported in rodents [172,177] and rhesus monkeys [176]. MBL-A and MBL-C deficient mice were found to be more susceptible to *S. aureus* infection [178]. Similar oligomerisation profiles and circulating concentrations of MBL-A and MBL-C (5 to 40 μg/mL) were observed in murine laboratory strains [172]. However, MBL-C was found to be approximately one-fifth less functional than MBL-A in triggering complement activation in vitro [172]. Furthermore, MBL-A showed a greater affinity for alpha-methyl-d -glucose and d -glucose when compared to MBL-C [172]. MBL can interact with a wide range of viral pathogens in a complement-activation dependent as well as independent manner [170,179]. MBL can bind directly to retroviruses and influenza viruses via its CRD region [180,181,182]. Enhanced MBL levels in the lungs during IAV infection appear to have a protective role against IAV [180,183] through inhibition of viral hemagglutination, aggregation, and opsonisation of the viral particles in either a complement-dependent or independent manner [181,184,185]. The complement-independent effects of MBL against IAV are shared by other mammalian C-type lectins, like conglutinin, surfactant protein A (SP-A) and SP-D [181,186,187,188,189,190]. MBL directly binds HA and NA of IAV, thus, neutralising the viral particles [184]. However, certain IAV subtypes are resistant to MBL, which is primarily dependent on the degree of glycosylation on the HA globular region [180,191,192]. MBL^−/−^ mice show increased susceptibility to IAV infection from highly glycosylated viral strains of IAV compared to wild-type (WT) mice [193]. However, H1N1 and avian influenza A H9N2 strain showed enhanced production of pro-inflammatory response in WT mice compared to MBL^−/−^ mice, suggesting that MBL may also have an adverse effect against some strains of IAV infection [194].

MBL interacts with HIV-1 via its N-linked glycosylated envelope glycoprotein, gp120 [195]. HIV-1 can also evade adaptive immune responses through ‘glycan shielding’ in which mutations found in the gp120 glycosylation site can restrict the binding of neutralising antibody but can maintain its attachment with its cellular receptors [196]. Additionally, in vivo studies have suggested that MBL can interact with primary isolates of HIV-1 via carbohydrates structures found on gp120 or gp41 viral glycoproteins [195]. Saifuddin et al. have produced HIV-1 viral particles containing no gp120/gp41 and determined their respective binding with MBL by comparing them with gp120/gp41 positive viral particles. It was found that approximately five times HIV virions bound to MBL in the presence of gp120/gp41, indicating the importance of carbohydrates for binding of HIV-1 to MBL [195]. In addition, in HIV-1-infected patients, it was shown that antibodies were capable of neutralising viral load, but mutation at N-linked glycosylation sites in the env gene led viral particles to escape from viral neutralisation [196]. In vivo studies using macaque models of HIV-1 infection have also supported the idea that N-linked glycans are crucial in viral escape from neutralisation [197,198,199].

Neutralisation of HIV-1 by MBL can be complement-independent, involving opsonisation to enhance phagocytosis by macrophages and dendritic cells (DCs) [200]. Interestingly, even at normal physiological serum levels, MBL does not neutralise HIV-1 infection through complement activation [201,202], suggesting greater importance of complement-independent mechanisms via MBL. Moreover, lower MBL levels have been associated with an increased risk of HIV-1 transmission, or progression to Acquired immunodeficiency syndrome (AIDS) [203,204,205].

MBL also employs viral glycoprotein-mediated complement-dependent or independent mechanisms, as seen in the case of IAV [184,193], severe acute respiratory syndrome coronavirus (SARS-CoV) [206,207], Dengue virus (DV), West Nile virus (WNV) [208,209], and hepatitis C virus (HCV) [210]. Curiously, the ability of MBL to contribute to the pathogenesis of HIV-1 has been supported by the data showing that MBL can mediate enhancement of HIV-1 dissemination to the brain by gp120 via the chemokine receptor, CXCR4 [211,212]. Furthermore, increased MBL expression in HIV-1 infected brain can possibly suggest that MBL may cause neuroinflammation and neuronal injury via activation of MBL-mediated lectin pathway [175].

Genetic polymorphisms found in the MBL gene are associated with the progression of liver disease as a cause of chronic HBV and HCV infection [213,214,215,216,217]. However, this data conflicts with another study that suggests no link between MBL mutations and HBV infection [218]. MBL can directly bind E1 and E2 glycoproteins of HCV and trigger complement activation via MASP-2, causing neutralisation of HCV particles [210]. Furthermore, MBL-HCV binding was found to be sufficient to trigger the complement system via C4b deposition. Moreover, complement was reported to enhance antibody neutralisation of HCV particles [219], possibly indicating that MBL-mediated deposition of complement may be involved in the elimination of viral particles. MBL can also bind HBsAg (hepatitis B surface antigen) or N-linked glycosylated forms [210], but it is poorly studied whether this interaction neutralises the viral infectivity. MBL-HBsAg interaction can also result in complement activation, as well as enhanced C4 deposition [220]. In vivo studies using mice have reported that MBL seems to modulate immune responses to herpes simplex virus 2 (HSV-2) [221], as evident from the induction of type 1 INF-α and IFN-β [222]. Additionally, in vivo studies have suggested that certain immune cells were found to contribute to the innate immune responses to HSV infection [222]. NK cells were involved in anti-HSV immunity by triggering cytokine production, recognition, and killing infected cells [223,224]. Plasmacytoid dendritic cells (pDCs) were involved in type I IFN production in vivo [223,225]. Mice with MBL deficiency appear to be susceptible to recurrent infection of *Staphylococcus aureus* [178] and HSV-2 [226].

Higher susceptibility to SARS infection is reported in MBL-deficient individuals. Reduced serum levels of MBL were observed in patients infected with SARS-CoV infection [206,207]. MBL can bind and inhibit SARS-CoV through its CRD region in a complement-independent manner [207]. Previous studies have reported that spike protein (S) of SARS-CoV binds to DC-SIGN and DC-SIGNR [227,228], leading to enhanced viral infectivity; this can be inhibited by MBL binding with S protein SARS-CoV, and thereby blocking viral interaction with DC-SIGN [206]. DC-SIGN-R, also known as L-SIGN, a DC-SIGN homologue, has also been suggested to act as a direct receptor for SARS-CoV entry into its host cells, including type II alveolar and endothelial cells [229]. Therefore, the ability of MBL to interfere with SARS-CoV interaction with surface-bound C-type lectin receptors on these cells may restrict viral spread and pathogenicity. Both DC-SIGN and DC-SIGNR can bind to the receptor-binding domain (RBD) of the SARS-CoV-2 spike protein and mediate viral entry in endothelial cells [230]. Furthermore, DC-SIGNR can also interact with angiotensin-converting enzyme 2 (ACE2), a known cellular receptor for SARS-CoV-2 infection, suggesting a possible role for heterodimerization DC-SIGNR and ACE2 in SARS-CoV-2 entry and infection in cell types where both are present [230]. MBL can interact with Zaire Ebola Virus (EBOV) glycoprotein and prevent binding of Ebola and Marburg viruses to DC-SIGN, thus blocking its attachment to host cells [45]. In addition, using pseudotyped viral particles containing Ebola and Marburg (Musoke) glycoproteins, it was observed that MBL interaction with Ebola and Marburg viruses partially caused viral neutralisation via the lectin pathway [45]. Human serum deficient in MBL resulted in reduced neutralisation of pseudotyped viral particles with filoviruses, while the addition of MBL caused an enhanced neutralisation [45]. Furthermore, MBL induced cytokine storm by negating the activity of soluble glycoprotein [231]. MBL can potentially be involved in protection against enhanced vascular permeability, which is a known characteristic of Ebola haemorrhagic disease [231]. Mice administered with recombinant human MBL (rhMBL) showed a higher survival rate during fatal Ebola viral infection and became immune to viral re-infection [232]. Mice deficient in MBL-A, MBL-C, or MASP-2 were more vulnerable to WNV infection than wild-type mice, suggesting that MBL-mediated recognition and lectin pathway activation is vital for protection against WNV infection [233].

## 8. Ficolins

Ficolins are important innate immune PRRs, belonging to a group of oligomeric lectins, composed of N-terminal rich in cysteine residues, a collagen-like domain (CLD; composed of glycine-Xaa-Yaa repeats), and a neck region [234]. Like collectins, ficolins do not contain a CRD region, where it is replaced by a C-terminal fibrinogen-like (FBG) domain [235]. Like CRDs, the ficolins-derived FBG domain can also recognise specific pathogen-associated carbohydrates. Like MBL, homotrimers of ficolin are stabilised by the interactions between hydrophobic residues found in the CLD region [236,237] and oligomerise via both intermonomer and trimer disulphide bridges between the N-terminal cysteine residues [234,238].

In humans, three ficolins have been identified: M (ficolin-1), L (ficolin-2), and H-ficolin (ficolin-3) [239]. Only two ficolins have been identified in rodents, ficolin-A and ficolin-B, which are the orthologues of human L- and M-ficolin [130]. Both human L- and H-ficolins are expressed and secreted mainly by the hepatocytes [235,240], although type II alveolar and bronchial epithelial cells are also known to express higher levels of H-ficolins [240]. The crucial role of ficolins within the innate immunity is the recognition of pathogen-associated molecular patterns (PAMPs) on microbial pathogens by binding to acetylated polysaccharides (*N*-acetylglucosamine (GlcNAc) or *N*-acetylgalactosamine (GalNAc)) on microbial pathogens [241,242]. This is a common characteristic shared amongst all the ficolins discovered [243,244]. Furthermore, ficolins can bind to sialic acid, lipopolysaccharides, fungal 1,3-β-D-glucan, and bacterial peptidoglycan [245,246,247,248,249].

Like MBL, all ficolins can trigger the lectin pathway via MASP, induce phagocytosis via opsonisation, and stimulate the secretion of pro-inflammatory cytokines and nitric oxide by macrophages [250]. Human L-ficolin can bind HA and NA glycoproteins of IAV, and, neutralise viral infection and replication [251]. An in vitro study has reported that porcine plasma ficolin reduces the cytopathic effect and replication of porcine reproductive and respiratory syndrome virus in a GlcNAc-dependent manner [252]. Direct inhibition of IAV entry by L-ficolin has also been reported; it can promote complement-mediated lysis of IAV viral particles and of infected cells [251]. H-ficolin, purified from human serum and bronchoalveolar lavage fluid, can bind to IAV, thus blocking viral infectivity by inhibiting hemagglutination activity and viral aggregation and direct blocking of complement activation [253].

Interaction between L-ficolin and HCV triggers lysis of HCV infected cells via deposition of C4. However, L-ficolin interaction can be abrogated if the E2 glycoprotein of the HCV is not glycosylated [254]. Furthermore, a recombinant oligomeric L-ficolin was found to neutralise HCV entry in a human liver cell line, Huh7, in a dose-dependent manner [254,255]. This neutralisation was mediated by restricting E2 interaction with its cellular cell surface receptor, lipoprotein receptor and scavenger receptor B1, which are crucial for HCV entry into the host cells [254,255]. L-ficolin’s monomeric form is reported to activate complement [254] but cannot prevent HCV entry [256]. Furthermore, Ren et al. have suggested that L-ficolin can mediate complement activation following its interaction with gp120 of HIV-1 [241]. Human M-ficolin was also found to interact with pentraxin 3 (PTX3), which could potentiate immune responses against invading pathogens. M-ficolin interaction with long pentraxin, PTX3, was attributed to sialic acid, which triggers the lectin pathway [257]. In contrast, no complement activation was observed using the Y271F M-ficolin mutant. Interaction between M-ficolin and PTX3 was found to reduce the infectivity of IAV strains, PR-8, and Phil82 [253]. However, M-ficolin interaction with the Zaire Ebola virus glycoprotein’s mucin-like domain leads to increased viral infectivity of the host cells [258].

## 9. Conclusions

The important role of the complement system during viral infection cannot be overstated. It plays a critical role in determining the outcome of many viral infections. Further research in this area will help elucidate the complex mechanisms involved in the viral-host interaction and help develop improved therapeutics to combat viral infections. Furthermore, understanding the immune suppression mechanisms employed by viruses can help develop therapeutics.The complement-activation independent functions of several complement components against viral entry and cytokine storm appear to suggest the possibility of using recombinant form and fragments of complement inhibitors, as anti-viral therapy.

## Figures and Tables

**Table 1:** Interaction of complement proteins to viruses, and biological consequences.

Virus	C1q Binding	Consequences of C1q Binding	MBL Binding	Consequences of MBL Binding	C4BP Binding	Consequences of C4BP Binding	Properdin Binding	Consequences of Properdin Binding	Factor H Binding	Consequences of Factor H Binding
Murine Leukaemia Virus (MuLV)	+	Complement activation Viral lysis by human serum	?							
Human Immunodeficiency Virus (HIV-1, 2)	+	Complement activation No virolysis by human serum Enhancement of infection of C receptor-bearing cells	+	In vitro inhibition of the virus Complement activation Enhancement of infection Virus uptake by macrophages	?		+		+	Escape complement destruction
Human T Lymphotropic Virus (HTLV)	+	In vitro inhibition of the virus complement activation No virolysis by human serum	+							
Herpes Simplex Virus 1 (HSV-1)	+	Neutralisation of a gC null virus by a C1–C5 dependent mechanism	?							
Herpes Simplex Virus 2 (HSV-2)	?		+	Enhancement of infection in a murine model						
Human gamma-herpesvirus 8							+	Induces complement activation during de novo KSHV infection		
Epstein-Barr Virus (EBV)	+	Classical pathway activation No neutralisation of the virus Opsonisation of the virus	?							
Cytomegalovirus (CMV)	+	Classical pathway activation No lysis of infected cells	-							
Influenza A Virus (IAV)	+		+	C-independent virus inactivation Complement activation (guinea pig)	+	Differentially modulates the efficacy of viral entry and replication in a strain-dependent manner	+	Differentially modulates the efficacy of viral entry and replication in a strain-dependent manner	+	Differentially modulates the efficacy of viral entry and replication in a strain-dependent manner
Flavivirus					+	Viral NS1 limits complement activation by binding C4BP	+	Enhance complement activation		Enables immune evasion
Hepatitis B virus					+	Upregulates C4BPα through transcription factor Sp1 inhibiting complement activation				
Adenovirus					+	Increased uptake by hepatocytes Reduced hepatic and innate toxicity after systemic application of adenoviruses vector				
Sindbis virus									+	Reduced alternative pathway activation
SARS CoV 2	?	Lower C1q levels in the blood of severely ill patients Localised deposits of C1q in lungs suggesting classical pathway activation	?	N-protein of SARS-CoV-2 has been shown to interact with (MASP2)	?		?		?	The addition of factor H help mitigate damages caused by uncontrolled alternative pathway activation by viral S1/S2 protein

**Table 2:** Viral complement evasion proteins, their homologues and evasion mechanisms.

Virus Name	Viral Protein	Host Homologue	Targets	Evasion Mechanism
Herpesvirus Saimiri	Complement Control Protein Homologue (CCPH)	DAF, MCP	CP/LP; AP C3 convertase	Decay AccelerationCofactor Activity
Herpesvirus Saimiri	HVSCD59	CD59	C5b-8 and C5b-9	MAC Complex Inhibition
Kaposi’s Sarcoma-Associated Herpesvirus	KSHV Complement Control Protein (KCP)	DAF, MCP	CP/LP and AP C3 convertase	Decay AccelerationCofactor Activity
Murine Gamma-herpesvirus 68	γ-hv68 RCA Protein	DAF, MCP	CP/LP and AP C3 convertase	Exact mechanism unknown; Effect possibly mediated through C3 interaction
Variola Virus	Variola Virus Inhibitor Of Complement Enzymes (SPICE)	MCP	CP/LP and AP C3/C5 convertase	Decay AccelerationCofactor Activity
Vaccinia Virus	Vaccinia Virus Complement Control Protein (VCP)	MCP	CP/LP and AP C3/C5 convertase	Decay AccelerationCofactor Activity
Monkeypox Virus	Monkeypox Virus Inhibitor Of Complement Enzymes (MOPICE)	MCP	CP/LP C3 convertase	Cofactor Activity
Astrovirus	Coat Protein	Human Neutrophil Defensin-1	C1q and MBL	Classical Pathway Inactivation: Disassociates C1s from C1q Lectin Pathway Inactivation: Blocks Masp-2 interaction with MBL
Herpes Simplex Virus	Envelope Surface Glycoprotein C	N/A	AP C3 convertase and C3b	Blocks C3b interaction with properdin and C5 Accelerates decay of alternative pathway C3 Convertase
Hepatitis B virus	HBx protein	N/A	CD46, CD59 and C4BP α	MAC Complex Inhibition CP/LP C3 convertase
Influenza virus	Matrix Protein 1	N/A	C1q	Classical Pathway Inactivation: Inhibits C1q interaction with IgG antibodies
Dengue (DENV), West Nile Virus, And Yellow Fever (YFV)	Non-Structural Protein 1 (NS1)	N/A	C1s, C4, C9	Bind Factor H/C4BP → Cofactor Activity Decreases deposition of C3b and MAC Interacts with C4 And C1s → Reduces classical C3 convertase and reduced deposition of C4b And C3b Binds Clusterin & Vitronectin → MAC Complex Inhibition

## Data Availability

Not applicable.

## References

[1] Sim R.B., Tsiftsoglou S.A. (2004). Proteases of the complement system. Biochem. Soc. Trans..

[2] Müller-Eberhard H.J., Polley M.J., Calcott M.A. (1967). Formation and Functional Significance of a Molecular Complex Derived from the Second and the Fourth Component of Human Complement. J. Exp. Med..

[3] Naff G.B., Pensky J., Lepow I.H. (1964). The Macromolecular Nature of the First Component of Human Complement. J. Exp. Med..

[4] Polley M.J., Müller-Eberhard H.J. (1968). The second component of human complement: Its isolation, fragmentation by C’1 esterase, and incorporation into C’3 convertase. J. Exp. Med..

[5] Kishore U., Reid K.B. (1999). Modular organization of proteins containing C1q-like globular domain. Immunopharmacology.

[6] Kouser L., Abdul-Aziz M., Nayak A., Stover C.M., Sim R.B., Kishore U. (2013). Properdin and factor h: Opposing players on the alternative complement pathway “see-saw”. Front Immunol..

[7] Roos A., Bouwman L.H., van Gijlswijk-Janssen D.J., Faber-Krol M.C., Stahl G.L., Daha M.R. (2001). Human IgA Activates the Complement System Via the Mannan-Binding Lectin Pathway. J. Immunol..

[8] Malhotra R., Wormald M.R., Rudd P.M., Fischer P.B., Dwek R.A., Sim R.B. (1995). Glycosylation changes of IgG associated with rheumatooid arthritis can activate complement via the mannose-binding protein. Nat. Med..

[9] Stoermer K.A., Morrison T.E. (2011). Complement and viral pathogenesis. Virology.

[10] Lu J., Kishore U. (2017). C1 Complex: An Adaptable Proteolytic Module for Complement and Non-Complement Functions. Front Immunol..

[11] Woodruff T.M., Nandakumar K.S., Tedesco F. (2011). Inhibiting the C5–C5a receptor axis. Mol. Immunol..

[12] Muller-Eberhard H.J. (1986). The Membrane Attack Complex of Complement. Annu. Rev. Immunol..

[13] Manthey H.D., Woodruff T.M., Taylor S.M., Monk P.N. (2009). Complement component 5a (C5a). Int. J. Biochem. Cell Biol..

[14] Morgan B.P., McGeer P.L. (1995). Physiology and Pathophysiology of Complement: Progress and Trends. Crit. Rev. Clin. Lab. Sci..

[15] Klos A., Tenner A.J., Johswich K.-O., Ager R.R., Reis E.S., Köhl J. (2009). The role of the anaphylatoxins in health and disease. Mol. Immunol..

[16] Ember J., Jagels M., Hugli T. (1998). Characterization of complement anaphylatoxins and their biological responses. Hum. Complement Syst. Health Dis..

[17] Murakami Y., Imamichi T., Nagasawa S. (1993). Characterization of C3a anaphylatoxin receptor on guinea-pig macrophages. Immunology.

[18] Elsner J., Oppermann M., Czech W., Kapp A. (1994). C3a activates the respiratory burst in human polymorphonuclear neutrophilic leukocytes via pertussis toxin-sensitive G-proteins. Blood.

[19] Elsner J., Oppermann M., Czech W., Dobos G., Schöpf E., Norgauer J., Kapp A. (1994). C3a activates reactive oxygen radical species production and intracellular calcium transients in human eosinophils. Eur. J. Immunol..

[20] El-Lati S.G., Church M.K., Dahinden C.A. (1994). Complement Peptides C3a- and C5a-Induced Mediator Release from Dissociated Human Skin Mast Cells. J. Investig. Dermatol..

[21] Kretzschmar T., Jeromin A., Gietz C., Bautsch W., Klos A., Köhl J., Rechkemmer G., Bitter-Suermann D. (1993). Chronic myelogenous leukemia-derived basophilic granulocytes express a functional active receptor for the anaphylatoxin C3a. Eur. J. Immunol..

[22] Hartmann K., Henz B.M., Krüger-Krasagakes S., Köhl J., Burger R., Guhl S., Haase I., Lippert U., Zuberbier T. (1997). C3a and C5a Stimulate Chemotaxis of Human Mast Cells. Blood.

[23] Fischer W.H., Jagels M.A., Hugli T.E. (1999). Regulation of IL-6 Synthesis in Human Peripheral Blood Mononuclear Cells by C3a and C3a_desArg_. J. Immunol..

[24] Fischer W.H., Hugli T.E. (1997). Regulation of B cell functions by C3a and C3a(desArg): Suppression of TNF-alpha, IL-6, and the polyclonal immune response. J. Immunol..

[25] Peng Q., Li K., Sacks S.H., Zhou W. (2009). The role of anaphylatoxins C3a and C5a in regulating innate and adaptive immune responses. Inflamm. Allergy Drug Targets.

[26] Gorsuch W.B., Chrysanthou E., Schwaeble W.J., Stahl G.L. (2012). The complement system in ischemia–reperfusion injuries. Immunobiology.

[27] Farrar C., Asgari E., Schwaeble W., Sacks S. (2012). Which pathways trigger the role of complement in ischaemia/reperfusion injury?. Front. Immunol..

[28] Noris M., Remuzzi G. (2013). Overview of Complement Activation and Regulation. Semin. Nephrol..

[29] Janeway C. (1999). Immunobiology: The Immune System in Health and Disease.

[30] Chen J.Y., Cortes C., Ferreira V.P. (2018). Properdin: A multifaceted molecule involved in inflammation and diseases. Mol. Immunol..

[31] Ferreira V.P., Pangburn M.K., Cortes C. (2010). Complement control protein factor H: The good, the bad, and the inadequate. Mol. Immunol..

[32] Sim R.B., Day A.J., Moffatt B.E., Fontaine M. (1993). Complement factor I and cofactors in control of complement system convertase enzymes. Methods Enzym..

[33] Blom A.M., Kask L., Dahlbäck B. (2001). Structural Requirements for the Complement Regulatory Activities of C4BP *. J. Biol. Chem..

[34] Fujita T., Nussenzweig V. (1979). The role of C4-binding protein and {beta }1H in proteolysis of C4b and C3b. J. Exp. Med..

[35] Fujita T., Tamura N. (1983). Interaction of C4-binding protein with cell-bound C4b. A quantitative analysis of binding and the role of C4-binding protein in proteolysis of cell-bound C4b. J. Exp. Med..

[36] Lublin D.M., Atkinson J.P. (1989). Decay-Accelerating Factor: Biochemistry, Molecular Biology, and Function. Annu. Rev. Immunol..

[37] Liszewski M.K., Post T.W., Atkinson J.P. (1991). Membrane Cofactor Protein (MCP or CD46): Newest Member of the Regulators of Complement Activation Gene Cluster. Annu. Rev. Immunol..

[38] Ahearn J.M., Fearon D.T., Dixon F.J. (1989). Structure and Function of the Complement Receptors, CR1 (CD35) and CR2 (CD21). Advances in Immunology.

[39] Rollins S.A., Sims P.J. (1990). The complement-inhibitory activity of CD59 resides in its capacity to block incorporation of C9 into membrane C5b-9. J. Immunol..

[40] Berry D.M., Almeida J.D. (1968). The Morphological and Biological Effects of Various Antisera on Avian Infectious Bronchitis Virus. J. Gen. Virol..

[41] Cooper N. (1998). Complement and viruses. The Human Complement System in Health and Disease.

[42] Cooper N.R., Nemerow G.R. (1983). Complement, viruses, and virus-infected cells. Springer Semin. Immunopathol..

[43] Jayasekera J.P., Moseman E.A., Carroll M.C. (2007). Natural Antibody and Complement Mediate Neutralization of Influenza Virus in the Absence of Prior Immunity. J. Virol..

[44] Tam J.C.H., Bidgood S.R., McEwan W.A., James L.C. (2014). Intracellular sensing of complement C3 activates cell autonomous immunity. Science.

[45] Ji X., Olinger G.G., Aris S., Chen Y., Gewurz H., Spear G.T. (2005). Mannose-binding lectin binds to Ebola and Marburg envelope glycoproteins, resulting in blocking of virus interaction with DC-SIGN and complement-mediated virus neutralization. J. Gen. Virol..

[46] Blue C.E., Spiller O.B., Blackbourn D.J. (2004). The relevance of complement to virus biology. Virology.

[47] Ghannam A., Fauquert J.-L., Thomas C., Kemper C., Drouet C. (2014). Human complement C3 deficiency: Th1 induction requires T cell-derived complement C3a and CD46 activation. Mol. Immunol..

[48] Weaver D.J., Reis E.S., Pandey M.K., Köhl G., Harris N., Gerard C., Köhl J. (2010). C5a receptor-deficient dendritic cells promote induction of Treg and Th17 cells. Eur. J. Immunol..

[49] Barrington R.A., Schneider T.J., Pitcher L.A., Mempel T.R., Ma M., Barteneva N.S., Carroll M.C. (2009). Uncoupling CD21 and CD19 of the B-cell coreceptor. Proc. Natl. Acad. Sci. USA.

[50] Fodor W.L., Rollins S.A., Bianco-Caron S., Rother R.P., Guilmette E.R., Burton W.V., Albrecht J.C., Fleckenstein B., Squinto S.P. (1995). The complement control protein homolog of herpesvirus saimiri regulates serum complement by inhibiting C3 convertase activity. J. Virol..

[51] Singh A.K., Mullick J., Bernet J., Sahu A. (2006). Functional Characterization of the Complement Control Protein Homolog of Herpesvirus Saimiri: ARG-118 IS CRITICAL FOR FACTOR I COFACTOR ACTIVITIES. J. Biol. Chem..

[52] Albrecht J.C., Fleckenstein B. (1992). New member of the multigene family of complement control proteins in herpesvirus saimiri. J. Virol..

[53] Mullick J., Singh A.K., Panse Y., Yadav V., Bernet J., Sahu A. (2005). Identification of Functional Domains in Kaposica, the Complement Control Protein Homolog of Kaposi’s Sarcoma-Associated Herpesvirus (Human Herpesvirus 8). J. Virol..

[54] Mullick J., Bernet J., Singh A.K., Lambris J.D., Sahu A. (2003). Kaposi’s sarcoma-associated herpesvirus (human herpesvirus 8) open reading frame 4 protein (Kaposica) is a functional homolog of complement control proteins. J. Virol..

[55] Spiller O.B., Blackbourn D.J., Mark L., Proctor D.G., Blom A.M. (2003). Functional Activity of the Complement Regulator Encoded by Kaposi’s Sarcoma-associated Herpesvirus*. J. Biol. Chem..

[56] Russo J.J., Bohenzky R.A., Chien M.-C., Chen J., Yan M., Maddalena D., Parry J.P., Peruzzi D., Edelman I.S., Chang Y. (1996). Nucleotide sequence of the Kaposi sarcoma-associated herpesvirus (HHV8). Proc. Natl. Acad. Sci. USA.

[57] Kapadia S.B., Molina H., van Berkel V., Speck S.H., Virgin H.W. (1999). Murine Gammaherpesvirus 68 Encodes a Functional Regulator of Complement Activation. J. Virol..

[58] Agrawal P., Nawadkar R., Ojha H., Kumar J., Sahu A. (2017). Complement Evasion Strategies of Viruses: An Overview. Front. Microbiol..

[59] Rosengard A.M., Liu Y., Nie Z., Jimenez R. (2002). Variola virus immune evasion design: Expression of a highly efficient inhibitor of human complement. Proc. Natl. Acad. Sci. USA.

[60] Kotwal G.J., Moss B. (1988). Vaccinia virus encodes a secretory polypeptide structurally related to complement control proteins. Nature.

[61] Kotwal G., Isaacs S., McKenzie R., Frank M., Moss B. (1990). Inhibition of the complement cascade by the major secretory protein of vaccinia virus. Science.

[62] Sahu A., Isaacs S.N., Soulika A.M., Lambris J.D. (1998). Interaction of Vaccinia Virus Complement Control Protein with Human Complement Proteins: Factor I-Mediated Degradation of C3b to iC3b_1_ Inactivates the Alternative Complement Pathway. J. Immunol..

[63] McKenzie R., Kotwal G.J., Moss B., Hammer C.H., Frank M.M. (1992). Regulation of Complement Activity by Vaccinia Virus Complement-Control Protein. J. Infect. Dis..

[64] Vanderplasschen A., Mathew E., Hollinshead M., Sim R.B., Smith G.L. (1998). Extracellular enveloped vaccinia virus is resistant to complement because of incorporation of host complement control proteins into its envelope. Proc. Natl. Acad. Sci. USA.

[65] Bonaparte R.S., Hair P.S., Banthia D., Marshall D.M., Cunnion K.M., Krishna N.K. (2008). Human Astrovirus Coat Protein Inhibits Serum Complement Activation via C1, the First Component of the Classical Pathway. J. Virol..

[66] Hair P.S., Gronemus J.Q., Crawford K.B., Salvi V.P., Cunnion K.M., Thielens N.M., Arlaud G.J., Rawal N., Krishna N.K. (2010). Human astrovirus coat protein binds C1q and MBL and inhibits the classical and lectin pathways of complement activation. Mol. Immunol..

[67] Gronemus J.Q., Hair P.S., Crawford K.B., Nyalwidhe J.O., Cunnion K.M., Krishna N.K. (2010). Potent inhibition of the classical pathway of complement by a novel C1q-binding peptide derived from the human astrovirus coat protein. Mol. Immunol..

[68] Lubinski J., Wang L., Mastellos D., Sahu A., Lambris J.D., Friedman H.M. (1999). In Vivo Role of Complement-Interacting Domains of Herpes Simplex Virus Type 1 Glycoprotein Gc. J. Exp. Med..

[69] Hung S.-L., Peng C., Kostavasili I., Friedman H.M., Lambris J.D., Eisenberg R.J., Cohen G.H. (1994). The interaction of glycoprotein C of herpes simplex virus types 1 and 2 with the alternative complement pathway. Virology.

[70] Kostavasili I., Sahu A., Friedman H.M., Eisenberg R.J., Cohen G.H., Lambris J.D. (1997). Mechanism of complement inactivation by glycoprotein C of herpes simplex virus. J. Immunol..

[71] Fries L.F., Friedman H.M., Cohen G.H., Eisenberg R.J., Hammer C.H., Frank M.M. (1986). Glycoprotein C of herpes simplex virus 1 is an inhibitor of the complement cascade. J. Immunol..

[72] Harris S.L., Frank I., Vee A., Cohen G.H., Eisenberg R.J., Friedman H.M. (1990). Glycoprotein C of Herpes Simplex Virus Type 1 Prevents Complement-Mediated Cell Lysis and Virus Neutralization. J. Infect. Dis..

[73] Rux A.H., Lou H., Lambris J.D., Friedman H.M., Eisenberg R.J., Cohen G.H. (2002). Kinetic Analysis of Glycoprotein C of Herpes Simplex Virus Types 1 and 2 Binding to Heparin, Heparan Sulfate, and Complement Component C3b. Virology.

[74] Chung K.M., Liszewski M.K., Nybakken G., Davis A.E., Townsend R.R., Fremont D.H., Atkinson J.P., Diamond M.S. (2006). West Nile virus nonstructural protein NS1 inhibits complement activation by binding the regulatory protein factor H. Proc. Natl. Acad. Sci. USA.

[75] Schlesinger J.J., Brandriss M.W., Putnak J.R., Walsh E.E. (1990). Cell surface expression of yellow fever virus non-structural glycoprotein NS1: Consequences of interaction with antibody. J. Gen. Virol..

[76] Winkler G., Maxwell S.E., Ruemmler C., Stollar V. (1989). Newly synthesized dengue-2 virus nonstructural protein NS1 is a soluble protein but becomes partially hydrophobic and membrane-associated after dimerization. Virology.

[77] Avirutnan P., Fuchs A., Hauhart R.E., Somnuke P., Youn S., Diamond M.S., Atkinson J.P. (2010). Antagonism of the complement component C4 by flavivirus nonstructural protein NS1. J. Exp. Med..

[78] Avirutnan P., Hauhart R.E., Somnuke P., Blom A.M., Diamond M.S., Atkinson J.P. (2011). Binding of Flavivirus Nonstructural Protein NS1 to C4b Binding Protein Modulates Complement Activation. J. Immunol..

[79] Kurosu T., Chaichana P., Yamate M., Anantapreecha S., Ikuta K. (2007). Secreted complement regulatory protein clusterin interacts with dengue virus nonstructural protein 1. Biochem. Biophys. Res. Commun..

[80] Conde J.N., da Silva E.M., Allonso D., Coelho D.R., Andrade I.d.S., de Medeiros L.N., Menezes J.L., Barbosa A.S., Mohana-Borges R. (2016). Inhibition of the Membrane Attack Complex by Dengue Virus NS1 through Interaction with Vitronectin and Terminal Complement Proteins. J. Virol..

[81] Kishore U., Gaboriaud C., Waters P., Shrive A.K., Greenhough T.J., Reid K.B., Sim R.B., Arlaud G.J. (2004). C1q and tumor necrosis factor superfamily: Modularity and versatility. Trends Immunol..

[82] Mortensen S.A., Sander B., Jensen R.K., Pedersen J.S., Golas M.M., Jensenius J.C., Hansen A.G., Thiel S., Andersen G.R. (2017). Structure and activation of C1, the complex initiating the classical pathway of the complement cascade. Proc. Natl. Acad. Sci. USA.

[83] Kishore U., Reid K.B. (2000). C1q: Structure, function, and receptors. Immunopharmacology.

[84] Reis E.S., Barbuto J.A., Isaac L. (2007). Complement components, regulators and receptors are produced by human monocyte-derived dendritic cells. Immunobiology.

[85] Castellano G., Woltman A.M., Nauta A.J., Roos A., Trouw L.A., Seelen M.A., Schena F.P., Daha M.R., van Kooten C. (2004). Maturation of dendritic cells abrogates C1q production in vivo and in vitro. Blood.

[86] Kaul M., Loos M. (2001). Expression of membrane C1q in human monocyte-derived macrophages is developmentally regulated and enhanced by interferon-gamma. FEBS Lett..

[87] Bulla R., Tripodo C., Rami D., Ling G.S., Agostinis C., Guarnotta C., Zorzet S., Durigutto P., Botto M., Tedesco F. (2016). C1q acts in the tumour microenvironment as a cancer-promoting factor independently of complement activation. Nat. Commun..

[88] Mozdzanowska K., Feng J., Eid M., Zharikova D., Gerhard W. (2006). Enhancement of neutralizing activity of influenza virus-specific antibodies by serum components. Virology.

[89] Zhang J., Li G., Liu X., Wang Z., Liu W., Ye X. (2009). Influenza A virus M1 blocks the classical complement pathway through interacting with C1qA. J. Gen. Virol..

[90] Thielens N.M., Tacnet-Delorme P., Arlaud G.J. (2002). Interaction of C1q and mannan-binding lectin with viruses. Immunobiology.

[91] Fausther-Bovendo H., Vieillard V., Sagan S., Bismuth G., Debre P. (2010). HIV gp41 engages gC1qR on CD4+ T cells to induce the expression of an NK ligand through the PIP3/H2O2 pathway. PLoS Pathog..

[92] Ebenbichler C.F., Thielens N.M., Vornhagen R., Marschang P., Arlaud G.J., Dierich M.P. (1991). Human immunodeficiency virus type 1 activates the classical pathway of complement by direct C1 binding through specific sites in the transmembrane glycoprotein gp41. J. Exp. Med..

[93] Thielens N.M., Bally I.M., Ebenbichler C.F., Dierich M.P., Arlaud G.J. (1993). Further characterization of the interaction between the C1q subcomponent of human C1 and the transmembrane envelope glycoprotein gp41 of HIV-1. J. Immunol..

[94] Kishore U., Gupta S.K., Perdikoulis M.V., Kojouharova M.S., Urban B.C., Reid K.B. (2003). Modular organization of the carboxyl-terminal, globular head region of human C1q A, B, and C chains. J. Immunol..

[95] Pinter A., Honnen W.J., Tilley S.A., Bona C., Zaghouani H., Gorny M.K., Zolla-Pazner S. (1989). Oligomeric structure of gp41, the transmembrane protein of human immunodeficiency virus type 1. J. Virol..

[96] Marschang P., Kruger U., Ochsenbauer C., Gurtler L., Hittmair A., Bosch V., Patsch J.R., Dierich M.P. (1997). Complement activation by HIV-1-infected cells: The role of transmembrane glycoprotein gp41. J. Acquir. Immune Defic. Syndr. Hum. Retrovirol..

[97] Stoiber H., Ebenbichler C., Schneider R., Janatova J., Dierich M.P. (1995). Interaction of several complement proteins with gp120 and gp41, the two envelope glycoproteins of HIV-1. AIDS.

[98] Stoiber H., Thielens N.M., Ebenbichler C., Arlaud G.J., Dierich M.P. (1994). The envelope glycoprotein of HIV-1 gp120 and human complement protein C1q bind to the same peptides derived from three different regions of gp41, the transmembrane glycoprotein of HIV-1, and share antigenic homology. Eur J. Immunol..

[99] Susal C., Kirschfink M., Kropelin M., Daniel V., Opelz G. (1996). Identification of complement activation sites in human immunodeficiency virus type-1 glycoprotein gp120. Blood.

[100] Aasa-Chapman M.M., Holuigue S., Aubin K., Wong M., Jones N.A., Cornforth D., Pellegrino P., Newton P., Williams I., Borrow P. (2005). Detection of antibody-dependent complement-mediated inactivation of both autologous and heterologous virus in primary human immunodeficiency virus type 1 infection. J. Virol..

[101] Pednekar L., Pandit H., Paudyal B., Kaur A., Al-Mozaini M.A., Kouser L., Ghebrehiwet B., Mitchell D.A., Madan T., Kishore U. (2016). Complement Protein C1q Interacts with DC-SIGN via Its Globular Domain and Thus May Interfere with HIV-1 Transmission. Front. Immunol..

[102] Szabo J., Cervenak L., Toth F.D., Prohaszka Z., Horvath L., Kerekes K., Beck Z., Bacsi A., Erdei A., Peerschke E.I. (2001). Soluble gC1q-R/p33, a cell protein that binds to the globular “heads” of C1q, effectively inhibits the growth of HIV-1 strains in cell cultures. Clin. Immunol..

[103] Kittlesen D.J., Chianese-Bullock K.A., Yao Z.Q., Braciale T.J., Hahn Y.S. (2000). Interaction between complement receptor gC1qR and hepatitis C virus core protein inhibits T-lymphocyte proliferation. J. Clin. Investig..

[104] Mohan K.V., Ghebrehiwet B., Atreya C.D. (2002). The N-terminal conserved domain of rubella virus capsid interacts with the C-terminal region of cellular p32 and overexpression of p32 enhances the viral infectivity. Virus Res..

[105] Matthews D.A., Russell W.C. (1998). Adenovirus core protein V interacts with p32--a protein which is associated with both the mitochondria and the nucleus. J. Gen. Virol..

[106] Wang Y., Finan J.E., Middeldorp J.M., Hayward S.D. (1997). P32/TAP, a cellular protein that interacts with EBNA-1 of Epstein-Barr virus. Virology.

[107] Vieillard V., Strominger J.L., Debre P. (2005). NK cytotoxicity against CD4+ T cells during HIV-1 infection: A gp41 peptide induces the expression of an NKp44 ligand. Proc. Natl. Acad. Sci. USA.

[108] Moretta A., Bottino C., Vitale M., Pende D., Cantoni C., Mingari M.C., Biassoni R., Moretta L. (2001). Activating receptors and coreceptors involved in human natural killer cell-mediated cytolysis. Annu. Rev. Immunol..

[109] Braun L., Ghebrehiwet B., Cossart P. (2000). gC1q-R/p32, a C1q-binding protein, is a receptor for the InlB invasion protein of Listeria monocytogenes. EMBO J..

[110] Spear G.T., Jiang H.X., Sullivan B.L., Gewurz H., Landay A.L., Lint T.F. (1991). Direct binding of complement component C1q to human immunodeficiency virus (HIV) and human T lymphotrophic virus-I (HTLV-I) coinfected cells. AIDS Res. Hum. Retrovir..

[111] Ikeda F., Haraguchi Y., Jinno A., Iino Y., Morishita Y., Shiraki H., Hoshino H. (1998). Human complement component C1q inhibits the infectivity of cell-free HTLV-I. J. Immunol..

[112] Sagara Y., Inoue Y., Shiraki H., Jinno A., Hoshino H., Maeda Y. (1996). Identification and mapping of functional domains on human T-cell lymphotropic virus type 1 envelope proteins by using synthetic peptides. J. Virol..

[113] Bartholomew R.M., Esser A.F., Muller-Eberhard H.J. (1978). Lysis of oncornaviruses by human serum. Isolation of the viral complement (C1) receptor and identification as p15E. J. Exp. Med..

[114] Kunnakkadan U., Nag J., Kumar N.A., Mukesh R.K., Suma S.M., Johnson J.B. (2019). Complement-Mediated Neutralization of a Potent Neurotropic Human Pathogen, Chandipura Virus, Is Dependent on C1q. J. Virol..

[115] Blom A.M. (2002). Structural and functional studies of complement inhibitor C4b-binding protein. Biochem. Soc. Trans..

[116] Norman D.G., Barlow P.N., Baron M., Day A.J., Sim R.B., Campbell I.D. (1991). Three-dimensional structure of a complement control protein module in solution. J. Mol. Biol..

[117] Blom A.M., Villoutreix B.O., Dahlbäck B. (2003). Mutations in α-Chain of C4BP That Selectively Affect Its Factor I Cofactor Function*. J. Biol. Chem..

[118] Blom A.M., Webb J., Villoutreix B.O., Dahlbäck B. (1999). A Cluster of Positively Charged Amino Acids in the C4BP α-Chain Is Crucial for C4b Binding and Factor I Cofactor Function*. J. Biol. Chem..

[119] Feng G., Li J., Zheng M., Yang Z., Liu Y., Zhang S., Ye L., Zhang W., Zhang X. (2016). Hepatitis B virus X protein up-regulates C4b-binding protein α through activating transcription factor Sp1 in protection of hepatoma cells from complement attack. Oncotarget.

[120] Shan C., Zhang S., Cui W., You X., Kong G., Du Y., Qiu L., Ye L., Zhang X. (2011). Hepatitis B virus X protein activates CD59 involving DNA binding and let-7i in protection of hepatoma and hepatic cells from complement attack. Carcinogenesis.

[121] Zhang S., Shan C., Cui W., You X., Du Y., Kong G., Gao F., Ye L., Zhang X. (2013). Hepatitis B virus X protein protects hepatoma and hepatic cells from complement-dependent cytotoxicity by up-regulation of CD46. FEBS Lett..

[122] Shayakhmetov D.M., Gaggar A., Ni S., Li Z.-Y., Lieber A. (2005). Adenovirus Binding to Blood Factors Results in Liver Cell Infection and Hepatotoxicity. J. Virol..

[123] Christiansen D., Devaux P., Réveil B., Evlashev A., Horvat B., Lamy J., Rabourdin-Combe C., Cohen J.H.M., Gerlier D. (2000). Octamerization Enables Soluble CD46 Receptor To Neutralize Measles Virus In Vitro and In Vivo. J. Virol..

[124] Varghese P.M., Murugaiah V., Beirag N., Temperton N., Khan H.A., Alrokayan S.H., Al-Ahdal M.N., Nal B., Al-Mohanna F.A., Sim R.B. (2021). C4b Binding Protein Acts as an Innate Immune Effector Against Influenza A Virus. Front. Immunol..

[125] Coleman M., Murray J., Willard H., Nolan K., Reid K., Blake D.J., Lindsay S., Bhattacharya S., Wright A., Davies K. (1991). Genetic and physical mapping around the properdin P gene. Genomics.

[126] Smith C., Pangburn M., Vogel C., Müller-Eberhard H. (1984). Molecular architecture of human properdin, a positive regulator of the alternative pathway of complement. J. Biol. Chem..

[127] Pangburn M. (1989). Analysis of the natural polymeric forms of human properdin and their functions in complement activation. J. Immunol..

[128] De Paula P.F., Barbosa J., Junior P., Ferriani V.P.L., Latorre M., Nudelman V., Isaac L. (2003). Ontogeny of complement regulatory proteins–concentrations of factor h, factor I, c4b-binding protein, properdin and vitronectin in healthy children of different ages and in adults. Scand. J. Immunol..

[129] Smith K.F., Nolan K.F., Reid K.B., Perkins S.J. (1991). Neutron and X-ray scattering studies on the human complement protein properdin provide an analysis of the thrombospondin repeat. Biochemistry.

[130] Perdikoulis M.V., Kishore U., Reid K.B. (2001). Expression and characterisation of the thrombospondin type I repeats of human properdin. Biochim. Et Biophys. Acta (Bba)-Protein Struct. Mol. Enzymol..

[131] Hourcade D.E. (2006). The role of properdin in the assembly of the alternative pathway C3 convertases of complement. J. Biol. Chem..

[132] Stover C.M., Luckett J.C., Echtenacher B., Dupont A., Figgitt S.E., Brown J., Männel D.N., Schwaeble W.J. (2008). Properdin plays a protective role in polymicrobial septic peritonitis. J. Immunol..

[133] Wirthmueller U., Dewald B., Thelen M., Schäfer M., Stover C., Whaley K., North J., Eggleton P., Reid K., Schwaeble W.J. (1997). Properdin, a positive regulator of complement activation, is released from secondary granules of stimulated peripheral blood neutrophils. J. Immunol..

[134] Schwaeble W., Dippold W.G., Schäfer M., Pohla H., Jonas D., Luttig B., Weihe E., Huemer H.P., Dierich M.P., Reid K. (1993). Properdin, a positive regulator of complement activation, is expressed in human T cell lines and peripheral blood T cells. J. Immunol..

[135] Schwaeble W., Huemer H.P., MÖST J., Dierich M.P., STRÖBEL M., Claus C., Reid K.B., ZIEGLER-HEITBROCK H.L. (1994). Expression of properdin in human monocytes. Eur. J. Biochem..

[136] Reis E., Barbuto J., Isaac L. (2006). Human monocyte-derived dendritic cells are a source of several complement proteins. Inflamm. Res..

[137] Linton S., Morgan B. (1999). Properdin deficiency and meningococcal disease—identifying those most at risk. Clin. Exp. Immunol..

[138] Kimura Y., Miwa T., Zhou L., Song W.-C. (2008). Activator-specific requirement of properdin in the initiation and amplification of the alternative pathway complement. BloodJ. Am. Soc. Hematol..

[139] Kouser L., Abdul-Aziz M., Tsolaki A.G., Singhal D., Schwaeble W.J., Urban B.C., Khan H.A., Sim R.B., Kishore U. (2016). A recombinant two-module form of human properdin is an inhibitor of the complement alternative pathway. Mol. Immunol..

[140] Kemper C., Mitchell L.M., Zhang L., Hourcade D.E. (2008). The complement protein properdin binds apoptotic T cells and promotes complement activation and phagocytosis. Proc. Natl. Acad. Sci. USA.

[141] Saggu G., Cortes C., Emch H.N., Ramirez G., Worth R.G., Ferreira V.P. (2013). Identification of a novel mode of complement activation on stimulated platelets mediated by properdin and C3 (H2O). J. Immunol..

[142] Kemper C., Atkinson J.P., Hourcade D.E. (2009). Properdin: Emerging roles of a pattern-recognition molecule. Annu. Rev. Immunol..

[143] Jeon H., Yoo S.-M., Choi H.S., Mun J.Y., Kang H.-G., Lee J., Park J., Gao S.-J., Lee M.-S. (2017). Extracellular vesicles from KSHV-infected endothelial cells activate the complement system. Oncotarget.

[144] Dalrymple N.A., Mackow E.R. (2012). Endothelial Cells Elicit Immune-Enhancing Responses to Dengue Virus Infection. J. Virol..

[145] Stoiber H., Schneider R., Janatova J., Dierich M.P. (1995). Human complement proteins C3b, C4b, factor H and properdin react with specific sites in gpl20 and gp4l, the envelope proteins of HIV1. Immunobiology.

[146] Narni-Mancinelli E., Gauthier L., Baratin M., Guia S., Fenis A., Deghmane A.-E., Rossi B., Fourquet P., Escalière B., Kerdiles Y.M. (2017). Complement factor P is a ligand for the natural killer cell–activating receptor NKp46. Sci. Immunol..

[147] Beli E. (2012). Natural Killer Cell Responses to Influenza Virus Infection in Aged Mice. Ph.D. Thesis.

[148] Barrow A.D., Martin C.J., Colonna M. (2019). The natural cytotoxicity receptors in health and disease. Front. Immunol..

[149] Guzzo C., Fox J., Lin Y., Miao H., Cimbro R., Volkman B.F., Fauci A.S., Lusso P. (2013). The CD8-derived chemokine XCL1/lymphotactin is a conformation-dependent, broad-spectrum inhibitor of HIV-1. PLoS Pathog..

[150] Gupta S.S., Wang J., Chen M. (2020). Metabolic reprogramming in CD8+ T cells during acute viral infections. Front. Immunol..

[151] Decker P. (2011). Neutrophils and interferon-α-producing cells: Who produces interferon in lupus?. Arthritis Res. Ther..

[152] Kopp A., Hebecker M., Svobodova E., Jozsi M. (2012). Factor h: A complement regulator in health and disease, and a mediator of cellular interactions. Biomolecules.

[153] Fearon D.T. (1978). Regulation by membrane sialic acid of beta1H-dependent decay-dissociation of amplification C3 convertase of the alternative complement pathway. Proc. Natl. Acad. Sci. USA.

[154] Rodriguez de Cordoba S., Esparza-Gordillo J., Goicoechea de Jorge E., Lopez-Trascasa M., Sanchez-Corral P. (2004). The human complement factor H: Functional roles, genetic variations and disease associations. Mol. Immunol..

[155] Pangburn M.K., Pangburn K.L., Koistinen V., Meri S., Sharma A.K. (2000). Molecular mechanisms of target recognition in an innate immune system: Interactions among factor H, C3b, and target in the alternative pathway of human complement. J. Immunol..

[156] Gordon D.L., Kaufman R.M., Blackmore T.K., Kwong J., Lublin D.M. (1995). Identification of complement regulatory domains in human factor H. J. Immunol..

[157] McRae J.L., Duthy T.G., Griggs K.M., Ormsby R.J., Cowan P.J., Cromer B.A., McKinstry W.J., Parker M.W., Murphy B.F., Gordon D.L. (2005). Human factor H-related protein 5 has cofactor activity, inhibits C3 convertase activity, binds heparin and C-reactive protein, and associates with lipoprotein. J. Immunol..

[158] Zipfel P.F., Jokiranta T.S., Hellwage J., Koistinen V., Meri S. (1999). The factor H protein family. Immunopharmacology.

[159] DeCordova S., Abdelgany A., Murugaiah V., Pathan A.A., Nayak A., Walker T., Shastri A., Alrokayan S.H., Khan H.A., Singh S.K. (2019). Secretion of functionally active complement factor H related protein 5 (FHR5) by primary tumour cells derived from Glioblastoma Multiforme patients. Immunobiology.

[160] Hirsch R.L., Griffin D.E., Winkelstein J.A. (1981). Host modification of Sindbis virus sialic acid content influences alternative complement pathway activation and virus clearance. J. Immunol..

[161] Pinter C., Siccardi A.G., Longhi R., Clivio A. (1995). Direct interaction of complement factor H with the C1 domain of HIV type 1 glycoprotein 120. AIDS Res. Hum. Retrovir..

[162] Lambris J.D., Ricklin D., Geisbrecht B.V. (2008). Complement evasion by human pathogens. Nat. Rev. Microbiol..

[163] Brinton M.A. (2013). Replication cycle and molecular biology of the West Nile virus. Viruses.

[164] Colpitts T.M., Conway M.J., Montgomery R.R., Fikrig E. (2012). West Nile Virus: Biology, transmission, and human infection. Clin. Microbiol. Rev..

[165] Murugaiah V., Varghese P.M., Saleh S.M., Tsolaki A.G., Alrokayan S.H., Khan H.A., Collison K.S., Sim R.B., Nal B., Al-Mohanna F.A. (2020). Complement-Independent Modulation of Influenza A Virus Infection by Factor H. Front. Immunol..

[166] Kuhlman M., Joiner K., Ezekowitz R.A. (1989). The human mannose-binding protein functions as an opsonin. J. Exp. Med..

[167] Takahashi K., Chang W.-C., Takahashi M., Pavlov V., Ishida Y., La Bonte L., Shi L., Fujita T., Stahl G.L., Van Cott E.M. (2011). Mannose-binding lectin and its associated proteases (MASPs) mediate coagulation and its deficiency is a risk factor in developing complications from infection, including disseminated intravascular coagulation. Immunobiology.

[168] Takahashi K., Ip W.E., Michelow I.C., Ezekowitz R.A.B. (2006). The mannose-binding lectin: A prototypic pattern recognition molecule. Curr. Opin. Immunol..

[169] Murugaiah V., Tsolaki A.G., Kishore U. (2020). Collectins: Innate Immune Pattern Recognition Molecules. Adv. Exp. Med. Biol..

[170] Mason C.P., Tarr A.W. (2015). Human lectins and their roles in viral infections. Molecules.

[171] Turner M.W., Hamvas R.M. (2000). Mannose-binding lectin: Structure, function, genetics and disease associations. Rev. Immunogenet..

[172] Hansen S., Thiel S., Willis A., Holmskov U., Jensenius J.C. (2000). Purification and characterization of two mannan-binding lectins from mouse serum. J. Immunol..

[173] Sastry K., Zahedi K., Lelias J.M., Whitehead A.S., Ezekowitz R.A. (1991). Molecular characterization of the mouse mannose-binding proteins. The mannose-binding protein A but not C is an acute phase reactant. J. Immunol..

[174] Ezekowitz R.A., Day L.E., Herman G.A. (1988). A human mannose-binding protein is an acute-phase reactant that shares sequence homology with other vertebrate lectins. J. Exp Med.

[175] Singh K.K., Nathamu S., Adame A., Alire T.U., Dumaop W., Gouaux B., Moore D.J., Masliah E. (2011). Expression of mannose binding lectin in HIV-1-infected brain: Implications for HIV-related neuronal damage and neuroAIDS. Neurobehav. HIV Med..

[176] Mogues T., Ota T., Tauber A.I., Sastry K.N. (1996). Characterization of two mannose-binding protein cDNAs from rhesus monkey (Macaca mulatta): Structure and evolutionary implications. Glycobiology.

[177] Drickamer K., Dordal M.S., Reynolds L. (1986). Mannose-binding proteins isolated from rat liver contain carbohydrate-recognition domains linked to collagenous tails. Complete primary structures and homology with pulmonary surfactant apoprotein. J. Biol. Chem..

[178] Shi L., Takahashi K., Dundee J., Shahroor-Karni S., Thiel S., Jensenius J.C., Gad F., Hamblin M.R., Sastry K.N., Ezekowitz R.A. (2004). Mannose-binding lectin-deficient mice are susceptible to infection with Staphylococcus aureus. J. Exp. Med..

[179] Reading P.C., Hartley C.A., Ezekowitz R.A., Anders E.M. (1995). A serum mannose-binding lectin mediates complement-dependent lysis of influenza virus-infected cells. Biochem. Biophys. Res. Commun..

[180] Reading P.C., Morey L.S., Crouch E.C., Anders E.M. (1997). Collectin-mediated antiviral host defense of the lung: Evidence from influenza virus infection of mice. J. Virol..

[181] Hartshorn K.L., Sastry K., White M.R., Anders E.M., Super M., Ezekowitz R.A., Tauber A.I. (1993). Human mannose-binding protein functions as an opsonin for influenza A viruses. J. Clin. Investig..

[182] Hartley C.A., Jackson D.C., Anders E.M. (1992). Two distinct serum mannose-binding lectins function as beta inhibitors of influenza virus: Identification of bovine serum beta inhibitor as conglutinin. J. Virol..

[183] Fidler K.J., Hilliard T.N., Bush A., Johnson M., Geddes D.M., Turner M.W., Alton E.W., Klein N.J., Davies J.C. (2009). Mannose-binding lectin is present in the infected airway: A possible pulmonary defence mechanism. Thorax.

[184] Kase T., Suzuki Y., Kawai T., Sakamoto T., Ohtani K., Eda S., Maeda A., Okuno Y., Kurimura T., Wakamiya N. (1999). Human mannan-binding lectin inhibits the infection of influenza A virus without complement. Immunology.

[185] Anders E.M., Hartley C.A., Reading P.C., Ezekowitz R.A. (1994). Complement-dependent neutralization of influenza virus by a serum mannose-binding lectin. J. Gen. Virol..

[186] Al-Qahtani A.A., Murugaiah V., Bashir H.A., Pathan A.A., Abozaid S.M., Makarov E., Nal-Rogier B., Kishore U., Al-Ahdal M.N. (2019). Full-length human surfactant protein A inhibits influenza A virus infection of A549 lung epithelial cells: A recombinant form containing neck and lectin domains promotes infectivity. Immunobiology.

[187] Al-Ahdal M.N., Murugaiah V., Varghese P.M., Abozaid S.M., Saba I., Al-Qahtani A.A., Pathan A.A., Kouser L., Nal B., Kishore U. (2018). Entry Inhibition and Modulation of Pro-Inflammatory Immune Response Against Influenza A Virus by a Recombinant Truncated Surfactant Protein D. Front. Immunol..

[188] Crouch E., Hartshorn K., Horlacher T., McDonald B., Smith K., Cafarella T., Seaton B., Seeberger P.H., Head J. (2009). Recognition of mannosylated ligands and influenza A virus by human surfactant protein D: Contributions of an extended site and residue 343. Biochemistry.

[189] Hartshorn K.L., Webby R., White M.R., Tecle T., Pan C., Boucher S., Moreland R.J., Crouch E.C., Scheule R.K. (2008). Role of viral hemagglutinin glycosylation in anti-influenza activities of recombinant surfactant protein D. Respir. Res..

[190] Hartshorn K.L., Crouch E.C., White M.R., Eggleton P., Tauber A.I., Chang D., Sastry K. (1994). Evidence for a protective role of pulmonary surfactant protein D (SP-D) against influenza A viruses. J. Clin. Investig..

[191] Tokunaga H., Ushirogawa H., Ohuchi M. (2011). The pandemic (H1N1) 2009 influenza virus is resistant to mannose-binding lectin. Virol. J..

[192] Job E.R., Deng Y.M., Tate M.D., Bottazzi B., Crouch E.C., Dean M.M., Mantovani A., Brooks A.G., Reading P.C. (2010). Pandemic H1N1 influenza A viruses are resistant to the antiviral activities of innate immune proteins of the collectin and pentraxin superfamilies. J. Immunol..

[193] Chang W.C., White M.R., Moyo P., McClear S., Thiel S., Hartshorn K.L., Takahashi K. (2010). Lack of the pattern recognition molecule mannose-binding lectin increases susceptibility to influenza A virus infection. Bmc Immunol..

[194] Ling M.T., Tu W., Han Y., Mao H., Chong W.P., Guan J., Liu M., Lam K.T., Law H.K., Peiris J.S. (2012). Mannose-binding lectin contributes to deleterious inflammatory response in pandemic H1N1 and avian H9N2 infection. J. Infect. Dis..

[195] Saifuddin M., Hart M.L., Gewurz H., Zhang Y., Spear G.T. (2000). Interaction of mannose-binding lectin with primary isolates of human immunodeficiency virus type 1. J. Gen. Virol..

[196] Wei X., Decker J.M., Wang S., Hui H., Kappes J.C., Wu X., Salazar-Gonzalez J.F., Salazar M.G., Kilby J.M., Saag M.S. (2003). Antibody neutralization and escape by HIV-1. Nature.

[197] Cheng-Mayer C., Brown A., Harouse J., Luciw P.A., Mayer A.J. (1999). Selection for neutralization resistance of the simian/human immunodeficiency virus SHIVSF33A variant in vivo by virtue of sequence changes in the extracellular envelope glycoprotein that modify N-linked glycosylation. J. Virol..

[198] Reitter J.N., Means R.E., Desrosiers R.C. (1998). A role for carbohydrates in immune evasion in AIDS. Nat. Med..

[199] Chackerian B., Rudensey L.M., Overbaugh J. (1997). Specific N-linked and O-linked glycosylation modifications in the envelope V1 domain of simian immunodeficiency virus variants that evolve in the host alter recognition by neutralizing antibodies. J. Virol..

[200] Jack D.L., Lee M.E., Turner M.W., Klein N.J., Read R.C. (2005). Mannose-binding lectin enhances phagocytosis and killing of Neisseria meningitidis by human macrophages. J. Leukoc. Biol..

[201] Ying H., Ji X., Hart M.L., Gupta K., Saifuddin M., Zariffard M.R., Spear G.T. (2004). Interaction of mannose-binding lectin with HIV type 1 is sufficient for virus opsonization but not neutralization. AIDS Res. Hum. Retrovir..

[202] Senaldi G., Davies E.T., Mahalingam M., Lu J., Pozniak A., Peakman M., Reid K.B., Vergani D. (1995). Circulating levels of mannose binding protein in human immunodeficiency virus infection. J. Infect..

[203] Ballegaard V., Haugaard A.K., Garred P., Nielsen S.D., Munthe-Fog L. (2014). The lectin pathway of complement: Advantage or disadvantage in HIV pathogenesis?. Clin. Immunol..

[204] Takahashi K., Ezekowitz R.A. (2005). The role of the mannose-binding lectin in innate immunity. Clin. Infect. Dis..

[205] Garred P., Madsen H.O., Balslev U., Hofmann B., Pedersen C., Gerstoft J., Svejgaard A. (1997). Susceptibility to HIV infection and progression of AIDS in relation to variant alleles of mannose-binding lectin. Lancet.

[206] Zhou Y., Lu K., Pfefferle S., Bertram S., Glowacka I., Drosten C., Pohlmann S., Simmons G. (2010). A single asparagine-linked glycosylation site of the severe acute respiratory syndrome coronavirus spike glycoprotein facilitates inhibition by mannose-binding lectin through multiple mechanisms. J. Virol..

[207] Ip W.K., Chan K.H., Law H.K., Tso G.H., Kong E.K., Wong W.H., To Y.F., Yung R.W., Chow E.Y., Au K.L. (2005). Mannose-binding lectin in severe acute respiratory syndrome coronavirus infection. J. Infect. Dis..

[208] Avirutnan P., Hauhart R.E., Marovich M.A., Garred P., Atkinson J.P., Diamond M.S. (2011). Complement-mediated neutralization of dengue virus requires mannose-binding lectin. mBio.

[209] Fuchs A., Lin T.Y., Beasley D.W., Stover C.M., Schwaeble W.J., Pierson T.C., Diamond M.S. (2010). Direct complement restriction of flavivirus infection requires glycan recognition by mannose-binding lectin. Cell Host Microbe.

[210] Brown K.S., Keogh M.J., Owsianka A.M., Adair R., Patel A.H., Arnold J.N., Ball J.K., Sim R.B., Tarr A.W., Hickling T.P. (2010). Specific interaction of hepatitis C virus glycoproteins with mannan binding lectin inhibits virus entry. Protein Cell.

[211] Teodorof C., Divakar S., Soontornniyomkij B., Achim C.L., Kaul M., Singh K.K. (2014). Intracellular mannose binding lectin mediates subcellular trafficking of HIV-1 gp120 in neurons. Neurobiol. Dis..

[212] Bachis A., Aden S.A., Nosheny R.L., Andrews P.M., Mocchetti I. (2006). Axonal transport of human immunodeficiency virus type 1 envelope protein glycoprotein 120 is found in association with neuronal apoptosis. J. Neurosci..

[213] Hakozaki Y., Yoshiba M., Sekiyama K., Seike E., Iwamoto J., Mitani K., Mine M., Morizane T., Ohtani K., Suzuki Y. (2002). Mannose-binding lectin and the prognosis of fulminant hepatic failure caused by HBV infection. Liver.

[214] Sasaki K., Tsutsumi A., Wakamiya N., Ohtani K., Suzuki Y., Watanabe Y., Nakayama N., Koike T. (2000). Mannose-binding lectin polymorphisms in patients with hepatitis C virus infection. Scand. J. Gastroenterol..

[215] Yuen M.F., Lau C.S., Lau Y.L., Wong W.M., Cheng C.C., Lai C.L. (1999). Mannose binding lectin gene mutations are associated with progression of liver disease in chronic hepatitis B infection. Hepatology.

[216] Matsushita M., Hijikata M., Matsushita M., Ohta Y., Mishiro S. (1998). Association of mannose-binding lectin gene haplotype LXPA and LYPB with interferon-resistant hepatitis C virus infection in Japanese patients. J. Hepatol..

[217] Thomas H.C., Foster G.R., Sumiya M., McIntosh D., Jack D.L., Turner M.W., Summerfield J.A. (1996). Mutation of gene of mannose-binding protein associated with chronic hepatitis B viral infection. Lancet.

[218] Hohler T., Wunschel M., Gerken G., Schneider P.M., Meyer zum Buschenfelde K.H., Rittner C. (1998). No association between mannose-binding lectin alleles and susceptibility to chronic hepatitis B virus infection in German patients. Exp. Clin. Immunogenet..

[219] Meyer K., Basu A., Przysiecki C.T., Lagging L.M., Di Bisceglie A.M., Conley A.J., Ray R. (2002). Complement-mediated enhancement of antibody function for neutralization of pseudotype virus containing hepatitis C virus E2 chimeric glycoprotein. J. Virol..

[220] Chong W.P., To Y.F., Ip W.K., Yuen M.F., Poon T.P., Wong W.H., Lai C.L., Lau Y.L. (2005). Mannose-binding lectin in chronic hepatitis B virus infection. Hepatology.

[221] Gadjeva M., Paludan S.R., Thiel S., Slavov V., Ruseva M., Eriksson K., Lowhagen G.B., Shi L., Takahashi K., Ezekowitz A. (2004). Mannan-binding lectin modulates the response to HSV-2 infection. Clin. Exp. Immunol..

[222] Chew T., Taylor K.E., Mossman K.L. (2009). Innate and adaptive immune responses to herpes simplex virus. Viruses.

[223] Biron C.A., Brossay L. (2001). NK cells and NKT cells in innate defense against viral infections. Curr. Opin. Immunol..

[224] Cerwenka A., Lanier L.L. (2001). Ligands for natural killer cell receptors: Redundancy or specificity. Immunol. Rev..

[225] Lund J., Sato A., Akira S., Medzhitov R., Iwasaki A. (2003). Toll-like receptor 9-mediated recognition of Herpes simplex virus-2 by plasmacytoid dendritic cells. J. Exp. Med..

[226] Seppanen M., Lokki M.L., Lappalainen M., Hiltunen-Back E., Rovio A.T., Kares S., Hurme M., Aittoniemi J. (2009). Mannose-binding lectin 2 gene polymorphism in recurrent herpes simplex virus 2 infection. Hum. Immunol..

[227] Marzi A., Gramberg T., Simmons G., Moller P., Rennekamp A.J., Krumbiegel M., Geier M., Eisemann J., Turza N., Saunier B. (2004). DC-SIGN and DC-SIGNR interact with the glycoprotein of Marburg virus and the S protein of severe acute respiratory syndrome coronavirus. J. Virol..

[228] Yang Z.Y., Huang Y., Ganesh L., Leung K., Kong W.P., Schwartz O., Subbarao K., Nabel G.J. (2004). pH-dependent entry of severe acute respiratory syndrome coronavirus is mediated by the spike glycoprotein and enhanced by dendritic cell transfer through DC-SIGN. J. Virol..

[229] Jeffers S.A., Tusell S.M., Gillim-Ross L., Hemmila E.M., Achenbach J.E., Babcock G.J., Thomas W.D., Thackray L.B., Young M.D., Mason R.J. (2004). CD209L (L-SIGN) is a receptor for severe acute respiratory syndrome coronavirus. Proc. Natl. Acad. Sci. USA.

[230] Amraie R., Napoleon M.A., Yin W., Berrigan J., Suder E., Zhao G., Olejnik J., Gummuluru S., Muhlberger E., Chitalia V. (2020). CD209L/L-SIGN and CD209/DC-SIGN act as receptors for SARS-CoV-2 and are differentially expressed in lung and kidney epithelial and endothelial cells. bioRxiv.

[231] Escudero-Perez B., Volchkova V.A., Dolnik O., Lawrence P., Volchkov V.E. (2014). Shed GP of Ebola virus triggers immune activation and increased vascular permeability. PLoS Pathog..

[232] Michelow I.C., Lear C., Scully C., Prugar L.I., Longley C.B., Yantosca L.M., Ji X., Karpel M., Brudner M., Takahashi K. (2011). High-dose mannose-binding lectin therapy for Ebola virus infection. J. Infect Dis..

[233] Fuchs A., Pinto A.K., Schwaeble W.J., Diamond M.S. (2011). The lectin pathway of complement activation contributes to protection from West Nile virus infection. Virology.

[234] Hummelshoj T., Thielens N.M., Madsen H.O., Arlaud G.J., Sim R.B., Garred P. (2007). Molecular organization of human Ficolin-2. Mol. Immunol..

[235] Matsushita M., Endo Y., Taira S., Sato Y., Fujita T., Ichikawa N., Nakata M., Mizuochi T. (1996). A novel human serum lectin with collagen- and fibrinogen-like domains that functions as an opsonin. J. Biol. Chem..

[236] Weis W.I., Drickamer K. (1994). Trimeric structure of a C-type mannose-binding protein. Structure.

[237] Sheriff S., Chang C.Y., Ezekowitz R.A. (1994). Human mannose-binding protein carbohydrate recognition domain trimerizes through a triple alpha-helical coiled-coil. Nat. Struct. Biol..

[238] Ohashi T., Erickson H.P. (2004). The disulfide bonding pattern in ficolin multimers. J. Biol. Chem..

[239] Zhang X.L., Ali M.A. (2008). Ficolins: Structure, function and associated diseases. Adv. Exp. Med. Biol..

[240] Akaiwa M., Yae Y., Sugimoto R., Suzuki S.O., Iwaki T., Izuhara K., Hamasaki N. (1999). Hakata antigen, a new member of the ficolin/opsonin p35 family, is a novel human lectin secreted into bronchus/alveolus and bile. J. Histochem. Cytochem..

[241] Ren Y., Ding Q., Zhang X. (2014). Ficolins and infectious diseases. Virol. Sin..

[242] Matsushita M. (2013). Ficolins in complement activation. Mol. Immunol..

[243] Garlatti V., Belloy N., Martin L., Lacroix M., Matsushita M., Endo Y., Fujita T., Fontecilla-Camps J.C., Arlaud G.J., Thielens N.M. (2007). Structural insights into the innate immune recognition specificities of L- and H-ficolins. EMBO J..

[244] Liu Y., Endo Y., Iwaki D., Nakata M., Matsushita M., Wada I., Inoue K., Munakata M., Fujita T. (2005). Human M-ficolin is a secretory protein that activates the lectin complement pathway. J. Immunol..

[245] Swierzko A., Lukasiewicz J., Cedzynski M., Maciejewska A., Jachymek W., Niedziela T., Matsushita M., Lugowski C. (2012). New functional ligands for ficolin-3 among lipopolysaccharides of Hafnia alvei. Glycobiology.

[246] Honore C., Rorvig S., Hummelshoj T., Skjoedt M.O., Borregaard N., Garred P. (2010). Tethering of Ficolin-1 to cell surfaces through recognition of sialic acid by the fibrinogen-like domain. J. Leukoc. Biol..

[247] Ma Y.G., Cho M.Y., Zhao M., Park J.W., Matsushita M., Fujita T., Lee B.L. (2004). Human mannose-binding lectin and L-ficolin function as specific pattern recognition proteins in the lectin activation pathway of complement. J. Biol. Chem..

[248] Lynch N.J., Roscher S., Hartung T., Morath S., Matsushita M., Maennel D.N., Kuraya M., Fujita T., Schwaeble W.J. (2004). L-ficolin specifically binds to lipoteichoic acid, a cell wall constituent of Gram-positive bacteria, and activates the lectin pathway of complement. J. Immunol..

[249] Sugimoto R., Yae Y., Akaiwa M., Kitajima S., Shibata Y., Sato H., Hirata J., Okochi K., Izuhara K., Hamasaki N. (1998). Cloning and characterization of the Hakata antigen, a member of the ficolin/opsonin p35 lectin family. J. Biol. Chem..

[250] Luo F., Sun X., Wang Y., Wang Q., Wu Y., Pan Q., Fang C., Zhang X.L. (2013). Ficolin-2 defends against virulent Mycobacteria tuberculosis infection in vivo, and its insufficiency is associated with infection in humans. PLoS ONE.

[251] Pan Q., Chen H., Wang F., Jeza V.T., Hou W., Zhao Y., Xiang T., Zhu Y., Endo Y., Fujita T. (2012). L-ficolin binds to the glycoproteins hemagglutinin and neuraminidase and inhibits influenza A virus infection both in vitro and in vivo. J. Innate Immun..

[252] Keirstead N.D., Lee C., Yoo D., Brooks A.S., Hayes M.A. (2008). Porcine plasma ficolin binds and reduces infectivity of porcine reproductive and respiratory syndrome virus (PRRSV) in vitro. Antivir. Res..

[253] Verma A., White M., Vathipadiekal V., Tripathi S., Mbianda J., Ieong M., Qi L., Taubenberger J.K., Takahashi K., Jensenius J.C. (2012). Human H-ficolin inhibits replication of seasonal and pandemic influenza A viruses. J. Immunol..

[254] Liu J., Ali M.A., Shi Y., Zhao Y., Luo F., Yu J., Xiang T., Tang J., Li D., Hu Q. (2009). Specifically binding of L-ficolin to N-glycans of HCV envelope glycoproteins E1 and E2 leads to complement activation. Cell Mol. Immunol..

[255] Zhao Y., Ren Y., Zhang X., Zhao P., Tao W., Zhong J., Li Q., Zhang X.L. (2014). Ficolin-2 inhibits hepatitis C virus infection, whereas apolipoprotein E3 mediates viral immune escape. J. Immunol..

[256] Hamed M.R., Brown R.J., Zothner C., Urbanowicz R.A., Mason C.P., Krarup A., McClure C.P., Irving W.L., Ball J.K., Harris M. (2014). Recombinant human L-ficolin directly neutralizes hepatitis C virus entry. J. Innate Immun..

[257] Gout E., Moriscot C., Doni A., Dumestre-Perard C., Lacroix M., Perard J., Schoehn G., Mantovani A., Arlaud G.J., Thielens N.M. (2011). M-ficolin interacts with the long pentraxin PTX3: A novel case of cross-talk between soluble pattern-recognition molecules. J. Immunol..

[258] Favier A.L., Gout E., Reynard O., Ferraris O., Kleman J.P., Volchkov V., Peyrefitte C., Thielens N.M. (2016). Enhancement of Ebola Virus Infection via Ficolin-1 Interaction with the Mucin Domain of GP Glycoprotein. J. Virol..

